# Prevention of high-fat/high-sugar diet-induced type 2 diabetes mellitus-associated non-alcoholic fatty liver disease in rats with fermented and raw Rosa roxburghii Tratt (Cili) juice

**DOI:** 10.3389/fnut.2025.1584551

**Published:** 2025-05-19

**Authors:** Jingzhi Zhang, Haizhi Li, Huiru Yang, Liying Zhu, Yiqiong Zhang, Changyudong Huang, Shuang Wang, Yunfeng Duan, Zhaohui Jiang, Mi Liu, Shuyun Zhao, Wei Pan

**Affiliations:** ^1^The Key Laboratory of Environmental Pollution Monitoring and Disease Control, Ministry of Education, School of Public Health, Guizhou Medical University, Guiyang, China; ^2^Prenatal Diagnosis Center in Guizhou Province, The Affiliated Hospital of Guizhou Medical University, Guiyang, China; ^3^Department of Obstetrics and Gynecology, The Affiliated Hospital of Guizhou Medical University, Guiyang, China; ^4^Department of Nephrology, The Affiliated Hospital of Guizhou Medical University, Guiyang, China; ^5^School of Clinical Laboratory Science, Guizhou Medical University, Guiyang, China; ^6^The Clinical Laboratory Center, The First People’s Hospital of Guiyang, Guiyang, China; ^7^Molecular Pathology Laboratory, The Affiliated Cancer Hospital of Guizhou Medical University, Guiyang, China

**Keywords:** type 2 diabetes mellitus, non-alcoholic fatty liver disease, spontaneous fermented Cili juice, raw Cili juice, RNA sequencing, urine non-target metabolomics

## Abstract

**Introduction:**

Rosa roxburghii Tratt (Cili) is a medicinal and edible plant that has hypoglycemic, hypolipidemic, anti-inflammatory and antioxidant properties.

**Methods:**

To compare and confirm the potential effects of spontaneous fermented/raw Cili juice (FCJ/RCJ) on type 2 diabetes mellitus (T2DM), the constituents of FCJ and RCJ were characterized and compared, high-fat/high-sugar diet (HFD) and streptozocin (STZ)-induced T2DM-associated non-alcoholic fatty liver disease (NAFLD) model rats were gavaged with FCJ/RCJ (1.5 mL/kg/day) for 20 weeks, after which serum, urine and liver and pancreas tissues were collected for experiments, and urine non-targeted metabolomics and liver transcriptomics analyses were conducted, and enhanced analysis of metabolomics results and the liver transcriptome.

**Results:**

The findings demonstrated that FCJ and RCJ ameliorated hyperglycemia, dyslipidemia, and abnormal hepatic function; mitigated hepatic lipid accumulation; and restored insulin expression levels. These two kinds of Cili juices can modulate disorders of glucose and lipid metabolism, have anti-inflammatory effects, and improve sterol metabolism and bile acid homeostasis. Joint analysis indicated that the antidiabetic effects of FCJ/RCJ in rats with T2DM-associated NAFLD are mediated through improvements in insulin sensitivity, fatty acid metabolism, and bile acid homeostasis. Notably, FCJ has a stronger anti-inflammatory effect, whereas RCJ preferentially regulates bile acid homeostasis and mitochondrial β-oxidation.

**Conclusion:**

The in vivo experiments of T2DM-associated NAFLD rats conducted in this study demonstrate that FCJ/RCJ has the potential to ameliorate metabolic disorders.

## 1 Introduction

Type 2 diabetes mellitus (T2DM) is a complex chronic metabolic disorder characterized by hyperglycemia and insulin resistance (IR) that is difficult to reverse once diagnosed. T2DM is relatively complex and heterogeneous, affecting the pancreas and metabolic organs (1, 2). The liver serves as the largest metabolic organ in the human body, and hepatic dysfunction represents one of the prevalent complications associated with diabetes (3). The liver is highly sensitive to insulin and responds robustly to the intricate conditions induced by elevated blood glucose levels, potentially resulting in hepatocyte injury (4). Chronic dysregulation of glucose and lipid metabolism may have detrimental effects on hepatic function (5). There is a strong association between non-alcoholic fatty liver disease (NAFLD), T2DM and obesity, and T2DM and NAFLD share the same epidemiological and pathophysiological features (6). It has been suggested that up to 70% of patients with T2DM may also have NAFLD (7). The continuous exposure of patients with T2DM-associated NAFLD to hyperglycemia, IR, and excessive lipid accumulation can induce lipotoxicity and oxidative stress, which increases the risk of more severe liver damage, including cirrhosis, hepatocellular carcinoma, and even death (3). Therefore, liver damage is a late complication of diabetes. When T2DM is combined with NAFLD, the current research focuses primarily on recommending the most appropriate hypoglycemic treatment to potentially ameliorate NAFLD (8). However, there is a scarcity of drugs available for preventing and treating T2DM-associated NAFLD (9), thus necessitating more urgent research in this area.

Rosa roxburghii Tratt (also named Cili), a wild shrub plant of the genus Rosa, is native to Southwest China, mainly in Guizhou Province, and is an abundant plant used for both medicinal and food purposes. Cili contains a variety of nutrients, including vitamins, superoxide dismutase (SOD), flavonoid, terpenoids, polyphenols, polysaccharides, amino acids and trace elements. Encouragingly, recent studies have demonstrated the significant impact of Cili on glucose-lowering (10, 11), lipid-lowering (12), anti-inflammatory (13), antioxidant (14), immune-regulating (15), and other effects. Emerging evidence suggests that low molecular weight active metabolites- particularly polyphenols and flavonoids- exhibit superior bioavailability and direct or indirect modulatory effects on diabetes (16–18). Due to the high levels of tannins and organic acids, fresh Cili fruit has a sour taste and is acidic, so Cili is suitable for intensive processing. Fermentation is a highly efficient technique for improving the composition, flavor, and functionality of foods (19). Research has demonstrated that the fermentation of carrots by Lacticaseibacillus rhamnosus could substantially enhance the levels of free polyphenols and amino acids, and promoting the utilization of fructose to produce short-chain fatty acids (20). Several researchers have reported that the levels of vitamin C and SOD in fermented Cili juice are stable and not easily brown and that the quality of the product is stable (21). The unique fruity and ester aroma of the fermented juice alleviates the original pungent sour taste of fresh Cili fruit (22). Researchers have reported that the fermentation of Cili juice with a variety of probiotics and yeast improved hyperlipidemia in rats by restoring the imbalance of the intestinal flora to alleviate dyslipidemia and regulate the levels of cytokines in the spleens of the mice (23, 24). Studies have confirmed that the levels of active ingredients in fermented Cili juice (FCJ) are different from those in raw Cili juice (RCJ) (25). Several scholars have reported that the antioxidant capacity and α-glucosidase inhibitory activity of FCJ were significantly enhanced, but its cholesterol esterase inhibitory activity was markedly decreased (26). However, the majority of the aforementioned studies have utilized yeast or fermentation bacteria for Cili fermentation, few studies on the spontaneous fermentation of Cili rely on non-*Saccharomyces cerevisiae* yeast on the surface or inside of Cili fruit. Wild yeast can produce a variety of extracellular enzymes that affect the composition and flavor of Cili juice by acting on relevant substrates (27). We hypothesize that alterations in the components and composition of FCJ may influence its biological functions, resulting in different beneficial effects for patients with T2DM-associated NAFLD compared to those of RCJ. However, the specific mechanisms underlying these effects have not been adequately explored. Consequently, this study aimed to investigate how the antidiabetic effects of FCJ and RCJ (with particular attention to the changes of flavonoids and polyphenols detectable by Liquid Chromatography-Mass Spectrometry before and after fermentation) in T2DM-associated NAFLD rats, as well as their protective effects against concomitant liver damage. Through long-term postprandial administration of FCJ/RCJ in T2DM-associated NAFLD rats, we analyzed changes in relevant phenotypic parameters, serum liver function markers, lipid profiles, pathological alterations in both liver and pancreas tissues, urinary metabolite variations, and gene transcriptomics within the liver. This research aims to establish an experimental foundation for the development of Cili products for the prevention and management of T2DM-associated NAFLD.

## 2 Materials and methods

### 2.1 Reagents and antibodies

RCJ (Cili fruits were pressed directly and filtered) (No. 20210517) and FCJ (raw Cili juice spontaneously fermented for 300 days under constant temperature and pressure without oxygen) (28) (No. 20210520) were purchased from Shanwangguo Co., Ltd. (Guizhou, China), and Cili fruit were picked from Guizhou Province. The blood glucose meter and matched test strips were purchased from Sannuo Biosensing Co., Ltd. (Changsha, China). Insulin and C-P ELISA kits were purchased from Mlbio (Shanghai, China). STZ was purchased from Solarbio Science & Technology Co., Ltd. (Beijing, China). Total cholesterol (TC), triglyceride (TG), low-density lipoprotein cholesterol (LDL-C), aspartate aminotransferase (AST) and alanine aminotransferase (ALT) kits were obtained from Beckman Coulter (USA). Antibodies against insulin (GB11334) and horseradish peroxidase (HRP)-conjugated goat anti-rabbit antibody (G1213) were purchased from Servicebio Technology Co., Ltd. (Wuhan, China). Customized high-fat and high-sugar (HFD) chow (20% sucrose, 18% animal fat, 2% cholesterol, 0.2% bile salt, and 59.8% basic feed) was purchased from Double Lion Experimental Animal Feed Technology Co., Ltd. (Suzhou, China). The western blotting antibodies used included APOA2 (BM5624, BOSTER, China), IDI1 (A07892-1, BOSTER, China), ABCC3 (DF3874, Affinity, United States), β-tubulin (Solarbio, China), goat anti-rabbit (BS13278, Bioworld, United States), and goat anti-mouse (BS12478, Bioworld, United States) antibodies.

### 2.2 Determination of the total flavonoid, polyphenol contents and pH of Cili fermented and Raw juice

The analysis of total flavonoid and total polyphenol contents were performed using spectrophotometry (1530, Thermo Fisher Scientific, Finland), with rutin and gallic acid used as reference standards, according to Lian’s method (29). pH test strips were used to determine the pH value of the Cili juice purchased from Shanghai Xinsheng Co., Ltd. (Shanghai, China).

### 2.3 Ultrahigh-performance liquid chromatography/mass spectrum (UPLC/MS) analysis of fermented and raw Cili juice

FCJ and RCJ were replicated three times within each group. Each 100 μL Cili juice sample was pipetted into 400 μL of precooled methanol: acetonitrile (1:1, v/v), frozen at −20°C for 30 min, and then centrifuged at 12,000 rpm (4°C, 10 min). Then, 400 μL of the supernatant was concentrated to dryness under vacuum. Then, 150 μL of 50% methanol was added, the mixture was vortexed and centrifuged at 12,000 rpm (4°C, 10 min), and the sample supernatant was filtered through a 0.22 μm filter membrane. A total of 20 μL of each sample filtrate was mixed into a QC sample to evaluate instrument stability and data reliability. The supernatant was separated on a UPLC system (Thermo, Vanquish Flex, United States), and an ACQUITY UPLC HSS T3 column (100 Å, 1.8 μm, 2.1 mm × 100 mm) was used with a flow rate of 0.4 mL/min and an injection volume of 2 μL. To evaluate instrument stability and data reliability, 20 μL of each sample was mixed into a QC sample. A high-resolution mass spectrometer (Thermo, Orbitrap Exploris 120, United States) was subsequently used for metabolite detection in electrospray ionization (ESI) negative and positive ion modes. The QC sample was injected 2–4 times to stabilize the system prior to formal injections. During the injection process, one QC sample was injected every 5–10 samples for subsequent data evaluation and quality assurance.

The raw data were imported into the commercial software Compound Discoverer 3.3 (version 3.3.2.31; Thermo, Waltham, United States) for peak extraction, alignment, correction and other operations. Metabolite identification was performed using a self-built library, the PSNGM database, the mzCloud online library, HMDB, MoNA, LIPID MAPS, and the NIST_2020_MSMS spectral library. The correlation of substance expression levels among biological replicate samples within a group serves as a critical metric for evaluating the reliability of the experiment and the rationality of sample selection, Pearson correlation coefficient exceeding 0.8 indicates that the sample satisfies the experimental requirements. Principal component analysis (PCA) and orthogonal projections to latent structures discriminant analysis (OPLS-DA) were separately conducted using the R package Ropls for dimensionality reduction of the sample data. Metabolites with a *p* value < 0.05 and Variable importance for projection (VIP) > 1 were considered statistically significant. Clustering analysis of differential metabolite abundances was carried out using the Pheatmap package (version 1.0.12) in R to generate heatmaps and trend analysis plots. The functional analysis of the differentially abundant metabolites involved primarily the Kyoto Encyclopedia of Genes and Genomes (KEGG) database. This part of the study was assisted by Personalbio Co., Ltd. (Shanghai, China). UPLC/MS is suitable for the analysis of small molecule compounds. Consequently, the detection of polysaccharide components in FCJ and RCJ were not incorporated into this experiment.

### 2.4 Animals and treatment

The animal experiments were approved by the Guizhou Medical University Animal Ethics Committee (No. 2101266). A total of 24 male SD rats (200 ± 10 g, 6–7 weeks) were purchased from Liaoning Changsheng Biotechnology Co., Ltd., and housed in a room (temperature, 22 ± 2°C; humidity, 55–60%; and light/dark cycle, 12 h). All the animals had free access to food and water. After 7 days of adaptive feeding, the rats were randomly divided into 2 groups: the control group with 6 rats and the HFD group with 18 rats. The rats in the control group were fed an ordinary maintenance diet. Rats in the HFD group were fed a 12-week HFD, and blood from the eye sockets of seven randomly selected rats was collected to monitor the serum levels of glucose, insulin and C-peptide (C-P) at 0, 30, 60, 120, and 180 min after being fed 50% glucose oral solution (2 g/kg). The results of the oral glucose tolerance test (OGTT), insulin release experiment, C-P release experiment and HOMA-IR formula verified that IR was present in the HFD group (see Supplementary Figure 1). Next, the HFD group rats were intraperitoneally injected with 25 mg/kg STZ dissolved in 0.1 mol/L citric acid buffer (pH = 4.4) with HFD feeding, whereas the control group rats were intraperitoneally injected with an equal volume of citric acid buffer (pH = 4.4). After the STZ injecting, the fasting blood glucose (FBG) levels of the HFD group rats were measured several times in the following 2 weeks, FBG ≥ 7.1 mmol/L and random blood glucose ≥ 16.8 mmol/L were considered a successful T2DM rat model. After successful modeling, the rats in the HFD group were separated into the T2DM group, M-Cili-F group (T2DM rats treated with FCJ), and M-Cili-R group (T2DM rats treated with RCJ). The rats in the M-Cili-F and M-Cili-R groups were treated with FCJ or RCJ once a day (1.5 mL/kg/day) by gavage for 20 weeks, the experimental dose was determined according to the manufacturer’s recommended human oral dosage (Approximately 30-50 mL/day) and proportionally adjusted based on body surface area equivalence (30) (according to the preliminary experimental results, see Supplementary Figure 2). The rats in the control and T2DM groups were treated with the same volume of normal saline by gavage. All experimental gavage operations were performed at 6 p.m. every day after the animals had eaten. Body weight and FBG levels were monitored during the experiments. On the last day, after 12 h without food, the rats were anesthetized by intraperitoneal injection of sodium pentobarbital (100 mg/kg) and sacrificed by cervical dislocation, and liver and pancreas tissues and serum samples were obtained. The collected organs were either frozen at −80°C or immersed in 10% neutral formaldehyde fixative.

### 2.5 Monitoring of lipid accumulation levels in rat livers by ultrasound screening

The rats in the experimental groups were monitored by ultrasound at 15 weeks during the gavage procedure. After anesthesia was induced by isoflurane inhalation, the lipid accumulation level in the rat livers was examined using an animal ultrasound machine (Vetus7, Mindray, China). When lipid accumulation in the livers of the T2DM group rats was compared with that in the livers of the control group rats using ultrasound, it was confirmed that the T2DM rats had NAFLD. The animal model of T2DM-associated NAFLD was established according to the methods of Jianghua (31) and Jie (32).

### 2.6 Detection of serum biochemical indicators

Rat serum samples were tested using a Beckman AU5800 instrument and matched biochemical kits to measure the levels of TG, TC, LDL-C, AST, and ALT at the Affiliated Cancer Hospital of Guizhou Medical University.

### 2.7 Hematoxylin and eosin (H&E) and oil red O staining

Liver and pancreas samples fixed in 10% formalin solution were embedded in paraffin and sectioned. The experimental procedure for H&E staining was based on Zhang’s research (33). The sections were viewed under an optical microscope (Olympus, Japan). Oil Red O staining was used to evaluate hepatic fat accumulation. Fresh liver tissue was embedded in optimal cutting temperature (OCT) compound, and the OCT-embedded samples were sectioned at 4–5 μm and stained with Oil Red O for the evaluation of fat droplets under an optical microscope (Olympus, Japan).

### 2.8 Immunohistochemistry

β-cell mass in diabetes animal models is frequently evaluated by IHC. Paraffin-embedded pancreatic sections were deparaffinized, subjected to antigen retrieval, treated with 3% hydrogen peroxide, washed, and blocked with serum. The slides were incubated with an insulin antibody overnight at 4°C, and the next day, the secondary antibody was incubated at room temperature, followed by color development, nuclear staining with hematoxylin, dehydration, transparency, sealing, and final microscopic examination. Serial sections of pancreatic tissue with insulin immunoreactivity were evaluated for Insulin-positive areas (%).

### 2.9 UPLC/MS analysis of urine metabolites

The urine of each group of rats was collected using metabolic cages for a duration of 1 h in the morning, with volumes ranging from 500 to 1000 μL per rat. The collection was performed at a centrifugation speed of 1,000 rpm for 5 min at 4°C. The resulting urine supernatant was subsequently filtered through a 0.22 μm filter and stored at −80°C. Non-targeted metabolomics analysis was employed to identify differentially abundant metabolites in the urine samples from each rat group, with three replicates conducted for each group following the same methodology as described in section 4.3. This part of the study was assisted by Personalbio Co., Ltd. (Shanghai, China) (*n* = 3/group).

### 2.10 RNA sequencing

TRIzol reagent (Takara, Japan) was used to extract total RNA from the liver tissues of the rats according to the manufacturer’s instructions. A NanoDrop 2000 Ultra microspectrometer (Thermo Fisher, Durham, NC, United States) was used to detect the quantity and quality of the RNA samples. The total RNA samples were reverse transcribed with oligo-dT primers to produce a matched cDNA library. Successful library construction was confirmed by sequencing on an Illumina Nova 6000 (Illumina, United States). Finally, based on the RNA-Seq data, Personalbio Co., Ltd. (Shanghai, China) performed bioinformatic analyses of the differentially expressed genes (DEGs) between the groups. Twelve hepatic cDNA libraries were sequenced, and each group had three replicates. The DEGs were analyzed with the DESeq method, the screening conditions for significant expression differences were |log2FoldChange| > 1 and *p*-value < 0.05, and the different groups were compared. Personalbio Co., Ltd. (Shanghai, China) assisted with this segment of the experiment (*n* = 3/group).

### 2.11 Real-time fluorescence quantitative PCR

Real-time qPCR was performed using a SYBR TB Green kit (Takara, Japan) for relative quantification of gene expression on a CFX Connect Real-time System (Bio-Rad, Inc., United States). Tsingke Biotechnology Co., Ltd. (Beijing, China) designed and synthesized the rat primers (see Supplementary Table 1). We selected the rat hydroxymethylbilane synthase (*Hmbs*) gene as the housekeeping gene and used the 2^−ΔΔ^*^CT^* method to calculate the relative gene expression levels. To confirm the specificity of the expression of each gene, a melting curve with a single peak was generated for each experiment.

### 2.12 Western blotting

The liver tissues of the rats were lysed, homogenized and sonicated, the supernatants were centrifuged for 20 min at 4°C, and a BCA kit was used to detect the protein contents. Liver samples were separated by SDS–PAGE and transferred to 0.22 μm PVDF membranes for 1–2 h. The membranes were then incubated with primary antibodies overnight at 4°C and then with the corresponding secondary antibodies for 2 h at room temperature. The color was generated by adding a luminescent solution, and the relative protein expression levels were quantified using Image J.

### 2.13 Metabolome–transcriptome analysis

#### 2.13.1 Correlation analysis

Pearson correlation coefficients between genes of liver and components of FCJ/RCJ were computed using the core function in R. Correlation analysis was conducted on differentially expressed genes and differentially components. Pairs with Pearson correlation coefficients exceeding 0.8 were selected to construct correlation network diagrams.

#### 2.13.2 Two-way orthogonal PLS (O2PLS) analysis

All genes and metabolites were included in the O2PLS model. By utilizing the loading plot, variables with significant correlations and weights across different datasets were identified. Key variables that exerted notable influences on the other datasets were further screened. A loading plot of genes and metabolites exhibiting the highest correlation was generated. In this plot, each dot represents a gene or metabolite, and the magnitude of the absolute value in the coordinate system reflects the strength of its correlation with the other omics layer.

### 2.14 Statistical analysis

GraphPad Prism 9.5.1 (GraphPad Software, Inc., CA, United States) was used to construct graphs with the data and perform the statistical analysis. All the data were calculated and are expressed as the means ± SDs. An unpaired Student’s *t*-test was used to compare the differences between two groups when the data were normally distributed, and the variances were homogeneous. One-way ANOVA was used for multiple-group comparisons. Other cases that did not meet the conditions were tested using non-parametric tests. *p* < 0.05 was considered statistically significant.

## 3 Results

### 3.1 Analysis of the component contents of the FCJ and RCJ

The pH values of the FCJ and RCJ samples were between 3 and 4. The levels of total polyphenols and flavonoids in RCJ were significantly greater than those in FCJ (*p* < 0.0001) (see Table 1).

**TABLE 1:** Content of main components of fermented Cili juice and raw Cili juice.

Main components	Fermented Cili juice	Raw Cili juice	*p-*value
Total polyphenol (mg/mL)	10.51 ± 0.06	17.33 ± 0.18	<0.0001
Total flavonoid (mg/mL)	11.83 ± 0.07	17.20 ± 0.13^1^	<0.0001

^1^Data are presented as mean ± SD (*n* = 4).

**FIGURE 1:**
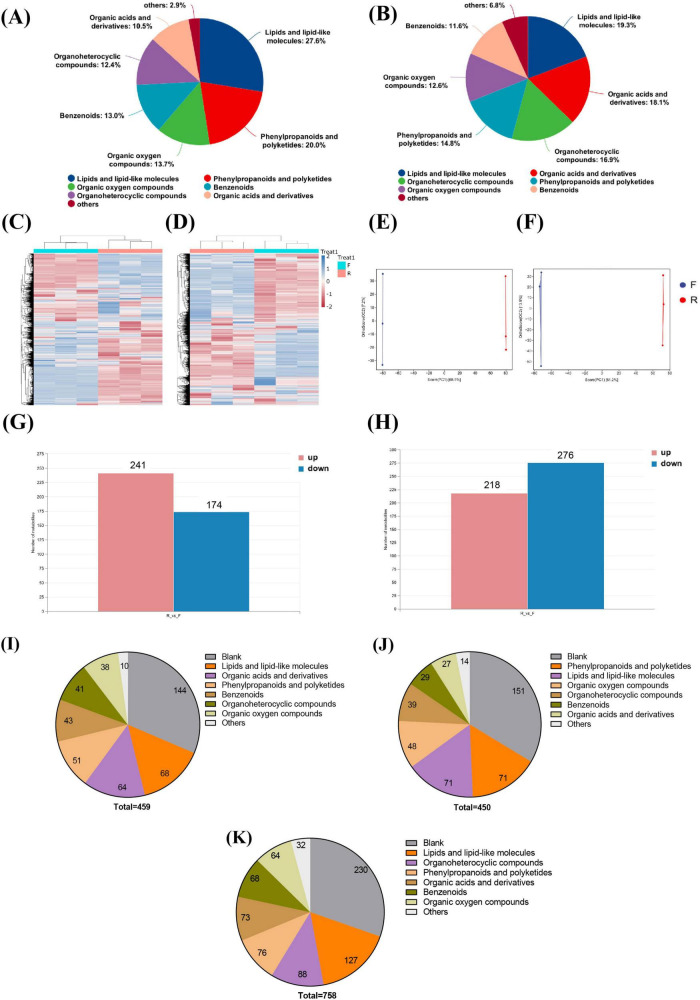
Analysis of differential components in FCJ and RCJ (*n* = 3). **(A,B)** The classification of all substances within the classes of both FCJ and RCJ in anion mode and cation mode. **(C,D)** Heatmaps of overall metabolites of FCJ and RCJ in anion mode and cation mode. **(E,F)** OPLS-DA score plots of FCJ and RCJ in anion mode and cation mode. **(G,H)** The numbers of differential components between FCJ and RCJ in anion mode and cation mode. **(I–K)** Classification of component composition in FCJ vs. RCJ. **(I)** Classification of substances with upregulated contents in FCJ compared with those in RCJ. **(J)** Classification of substances with downregulated contents in FCJ compared with those in RCJ. **(K)** Classification of substances with no significant differential content in FCJ compared with those in RCJ.

As observed in the total component PCA diagram in Supplementary Figures 3A,B, the repeated samples of FCJ, RCJ, and quality control (QC) were grouped together, demonstrating good repeatability of the samples. Upon analyzing all the substances detected in FCJ and RCJ, lipids and lipid-like molecules, organic acids and derivatives, and organo-heterocyclic compounds were found to be the main components of Cili juice in the cationic mode. In the anionic mode, lipids and lipid-like molecules, phenylpropanoids and polyketides, and organic oxygen compounds (13.7%) were the main components (Figures 1A,B). The overall metabolite heatmap revealed changes in the abundances of various components between RCJ and FCJ, as shown in Figures 1C,D. The OPLS-DA score plot revealed a significant disparity in composition between the two groups (Figures 1E,F). Compared with RCJ, FCJ had significantly increased contents of 241 components in anion mode and 218 components in cation mode, whereas 174 components were decreased in anion mode and 276 components were decreased in cation mode (Figures 1G,H).

Among the significantly elevated substances, in addition to the unclassified substances, lipids and lipid-like molecules, organic acids and derivatives, and phenylpropanoids and polyketides were predominant (see Figure 1I), and there were 32 flavonoids, polyphenols and vitamins with increased contents in FCJ, such as fisetin, luteolin, tyrosol, and pyridoxine. The primary components with significant decreases among the reduced substances were lipids and lipid-like molecules, excluding unclassified substances, phenylpropanoids and polyketides, and organic oxygen compounds dominated (see Figure 1J). Among these decreased compounds, 42 flavonoids, vitamins, and polyphenols were present in FCJ, including epicatechin, capsaicin, eriodyctiol and nicotinic acid. A total of 758 components did not significantly differ. In addition to unclassified substances, lipids and lipid-like molecules, organ heterocyclic compounds, and phenylpropanoid polyketides were found to be the predominant constituents (see Figure 1K), and there were 46 distinct flavonoids, vitamins and polyphenolic compounds, including quercetin, ascorbic acid, and rhamnetin, among others. Kyoto Encyclopedia of Genes and Genomes (KEGG) enrichment analysis was conducted to identify differential components between the two groups. Compared with those in RCJ, the primarily enriched substances in FCJ tended to upregulate metabolic pathways, such as the citrate cycle, glyoxylate and dicarboxylate metabolism, and pyruvate metabolism. Conversely, the enriched substances tended to downregulate cyan amino acid metabolism, valine/leucine/isoleucine biosynthesis, and galactose metabolism (see Supplementary Figure 3C).

### 3.2 Phenotypic effects of FCJ/RCJ in T2DM-associated NAFLD rats

**FIGURE 2:**
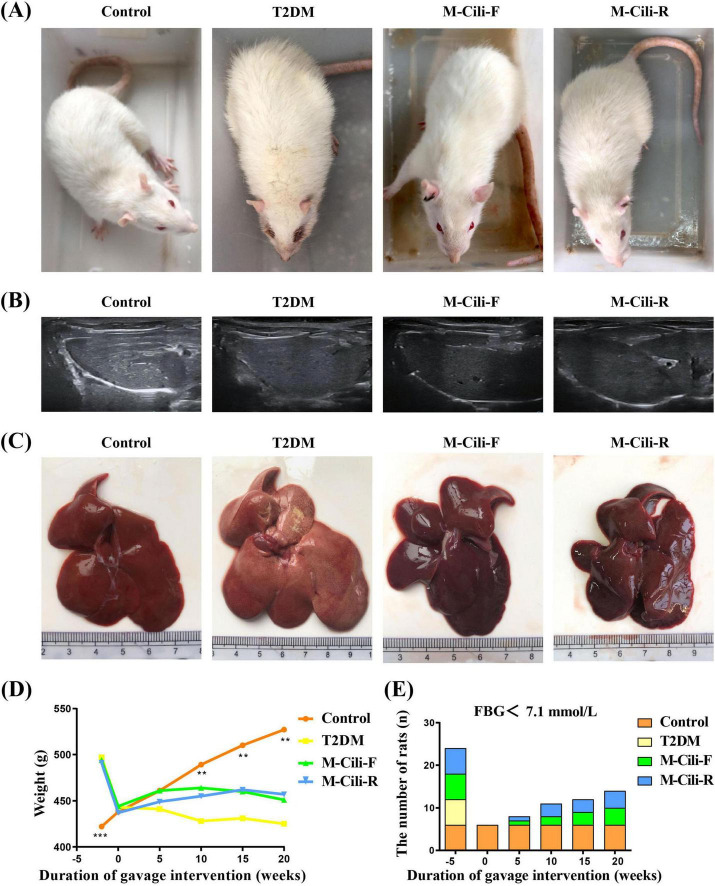
Rat phenotypes and liver morphology. **(A)** Phenotypes of the rats. **(B)** Representative images of the livers of the rats subjected to ultrasound scanning. **(C)** Phenotypes of the livers. **(D)** Changes in body weight. **(E)** Changes in the number of rats whose FBG returned to normal in each group after 20 weeks of intervention. Data are presented as mean ± SD (*n* = 6). *p < 0.05, **p < 0.01, ***p < 0.001, compared with T2DM group.

As shown in Figure 2, compared with the control group rats, the T2DM group rats presented significant weight loss at 20 weeks, yellow fur, and tiredness, whereas the fur color of the Cili juice-treated rats was glossier than that of the T2DM group rats (Figure 2A). Ultrasonographic changes in fat accumulation in the liver were also observed in the T2DM group but not in the control or intervention groups (see Figure 2B). In terms of the liver morphology of each group of rats, the livers of the control rats were reddish brown, smooth and soft, whereas the livers of the T2DM group rats were yellowish in color. The liver appearances of the M-Cili-F/R groups were similar to that of the control group (see Figure 2C). The weight loss of diabetic rats was significantly greater than that of control rats after the tenth week of modeling. Compared with that in the T2DM group, there was no significant difference in body weight between the M-Cili-F/R groups and the T2DM group, but the overall trends of body weight in the M-Cili-F and M-Cili-R groups were greater (Figure 2D). With increasing FCJ/RCJ intervention time in T2DM-associated NAFLD rats, the number of rats whose tail vein FBG values returned to normal gradually increased in the M-Cili-F/R groups (Figure 2E).

### 3.3 FCJ/RCJ improved serum liver function and lipid levels in T2DM-associated NAFLD rats

**FIGURE 3:**
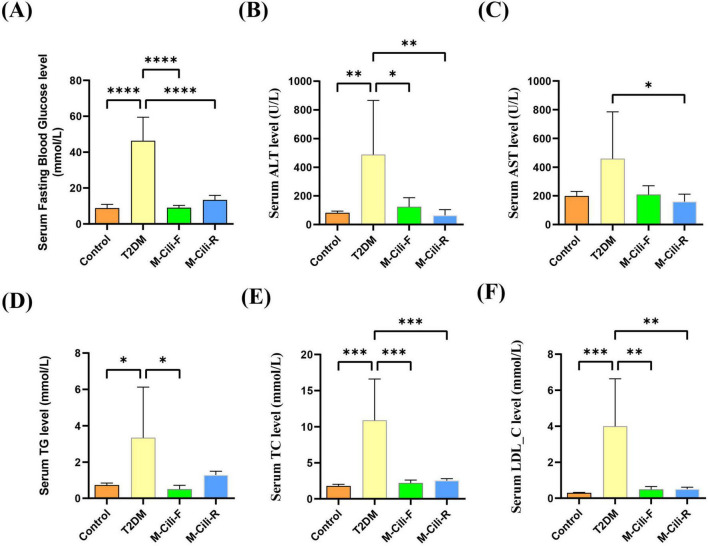
Biochemical indices of the rats in each group. **(A)** Serum FBG levels. **(B)** Serum ALT levels. **(C)** Serum AST levels. **(D)** Serum TG levels. **(E)** Serum TC levels. **(F)** Serum LDL-C levels. Data are presented as mean ± SD (*n* = 6). *p < 0.05, **p < 0.01, ***p < 0.001, ****p < 0.0001, compared with T2DM group.

At the end of the experiment, the serum FBG, ALT, TC, TG, and LDL-C levels were tested in the four groups. Compared with that in the control group, the level of serum FBG was significantly greater in the T2DM group (8.74 ± 2.18 mmol/L vs. 46.37 ± 13.11 mmol/L, *p* < 0.0001), whereas the levels of serum FBG in the M-Cili-F group (9.00 ± 1.33 mmol/L vs. 46.37 ± 13.11 mmol/L, *p* < 0.0001) and M-Cili-R group (13.28 ± 2.65 mmol/L vs. 46.37 ± 13.11 mmol/L, *p* < 0.0001) were significantly lower than that in the T2DM group. Compared with that in the control group, the level of serum ALT was significantly greater in the T2DM group (80.92 ± 12.86 U/L vs. 489.11 ± 376.96 U/L, *p* = 0.0075), while the levels of serum ALT in the M-Cili-F group (123.93 ± 63.15 U/L vs. 489.11 ± 376.96 U/L, *p* = 0.0177) and M-Cili-R group (63.44 ± 39.90 U/L vs. 489.11 ± 376.96 U/L, *p* = 0.0053) were significantly lower than that in the T2DM group. The level of serum TC was significantly greater in the T2DM group than that in the control group (1.78 ± 0.22 mmol/L vs. 10.89 ± 5.72 mmol/L, *p* = 0.0001), whereas the levels of serum TC in the M-Cili-F group (2.19 ± 0.41 mmol/L vs. 10.89 ± 5.72 mmol/L, *p* = 0.0002) and M-Cili-R group (2.53 ± 0.27 mmol/L vs. 10.89 ± 5.72 mmol/L, *p* = 0.0003) were significantly lower than that in the T2DM group. The level of serum LDL_C was significantly greater in the T2DM group than that in the control group (0.30 ± 0.03 mmol/L vs. 4.01 ± 2.63 mmol/L, *p* = 0.0006), whereas the levels of serum LDL_C in the M-Cili-F group (0.49 ± 0.16 mmol/L vs. 4.01 ± 2.63 mmol/L, *p* = 0.0010) and M-Cili-R group (0.50 ± 0.11 mmol/L vs. 4.01 ± 2.63 mmol/L, *p* = 0.0010) were significantly lower than that in the T2DM group (Figure 3). The results showed that FCJ/RCJ significantly alleviated hepatic damage and antihyperlipidemic effects in HFD-fed offspring.

### 3.4 FCJ/RCJ protected against liver injury induced in rats with T2DM-associated NAFLD

**FIGURE 4:**
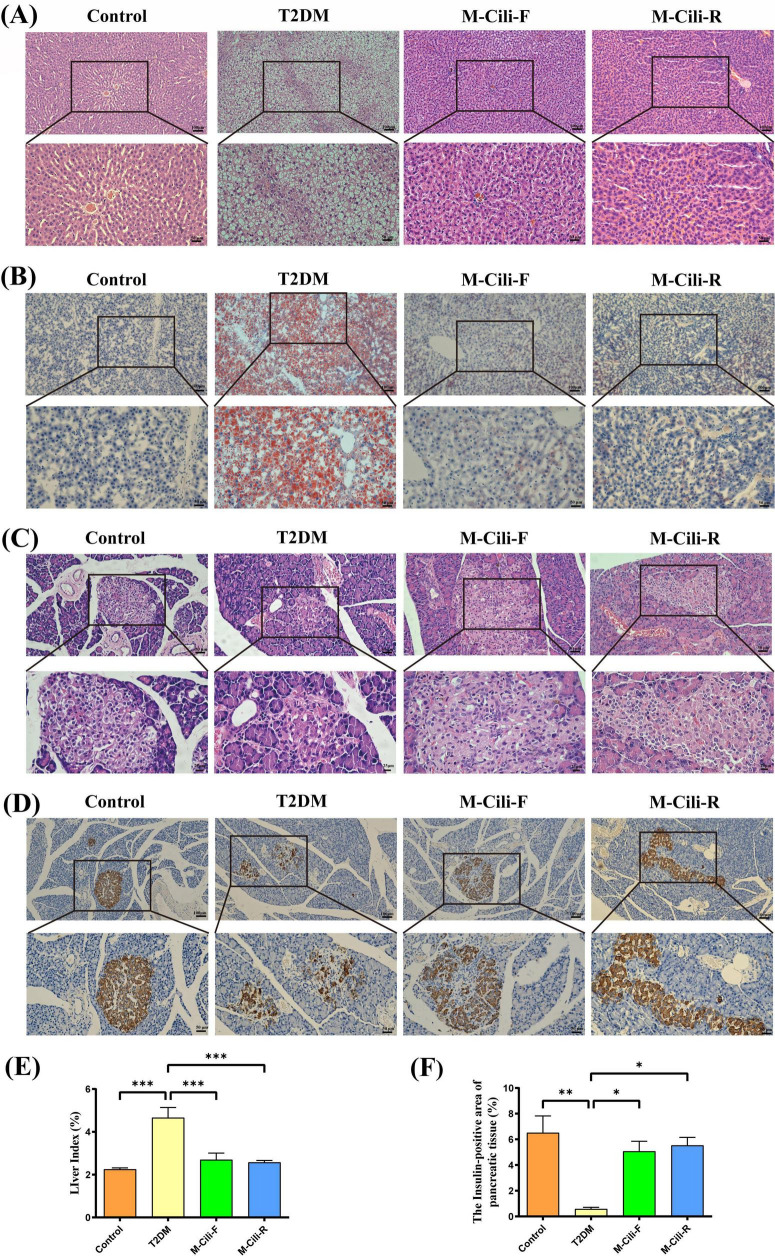
Morphology and staining of the liver and pancreas tissues of the rats. **(A)** H&E staining of the rat liver (100× and 200×). **(B)** Oil Red O staining of the rat liver (100× and 200×). **(C)** H&E staining of the pancreatic tissues of the rats (200× and 400×). **(D)** IHC of insulin expression in the pancreatic tissues of the rats (100× and 200×). **(E)** Liver index of the rats (%). **(F)** Area of positive insulin expression in the pancreas (%). Data are presented as mean ± SD (*n* = 6). *p < 0.05, **p < 0.01, ***p < 0.001, compared with T2DM group.

With respect to the liver tissue structure of the rats, the H&E staining results revealed that the control group presented well-structured liver lobules, well-arranged hepatocytes, and no hepatocyte steatosis. The rats in the T2DM group presented a disordered liver lobule structure, irregular hepatocyte morphology, obvious steatosis, and infiltration of multiple inflammatory cells. Compared with those in the T2DM group, both the M-Cili-F and M-Cili-R groups presented relatively clear and orderly cell structures, improved lipid accumulation, and significantly fewer inflammatory cells (see Figure 4A). Oil red O staining also revealed obvious lipid accumulation in the livers of the T2DM group, which was significantly improved after intervention with both Cili juices (Figure 4B). The liver index was significantly greater in the T2DM group than in the control group (2.09 ± 0.43 vs. 4.66 ± 1.17, *p* < 0.0001), whereas the liver indices were significantly lower in the M-Cili-F group (2.70 ± 0.76 vs. 4.66 ± 1.17, *p* = 0.0010) and M-Cili-R group (2.57 ± 0.22 vs. ± 4.66 ± 1.17, *p* = 0.0005) than in the T2DM group (see Figure 4E). The above results indicate that both FCJ and RCJ strongly prevent the abnormal lipid accumulation and liver damage induced in T2DM-associated NAFLD rats.

### 3.5 FCJ/RCJ protect the insulin secretion function of pancreatic tissue in T2DM-associated NAFLD rats

According to the H&E images of the pancreas, compared with that of the control group, the pancreatic cell area of the T2DM group was reduced, and the cell morphology was disordered. Compared with the T2DM group, the M-Cili-F and M-Cili-R groups presented more intact islet cell morphology (Figure 4C). According to the IHC results, compared with the control group, the T2DM group had a significantly reduced insulin-positive area (6.51 ± 3.20 vs. 0.58 ± 0.34, *p* = 0.0003). Compared with that of the T2DM group, the insulin-positive areas of the M-Cili-F and M-Cili-R groups significantly recovered (5.07 ± 1.92 vs. 0.58 ± 0.34, *p* = 0.0061; 5.53 ± 1.52 vs. 0.58 ± 0.34, *p* = 0.0024), which suggested that the expression level of Insulin was restored to a certain extent (Figures 4D,F). These results indicated that FCJ and RCJ had a protective effect on the ability of β cells to secrete insulin in the pancreas of T2DM-associated NAFLD rats.

### 3.6 FCJ/RCJ upregulates the levels of antidiabetic, bile acid and anti-inflammatory metabolites in rats with T2DM-associated NAFLD

**FIGURE 5:**
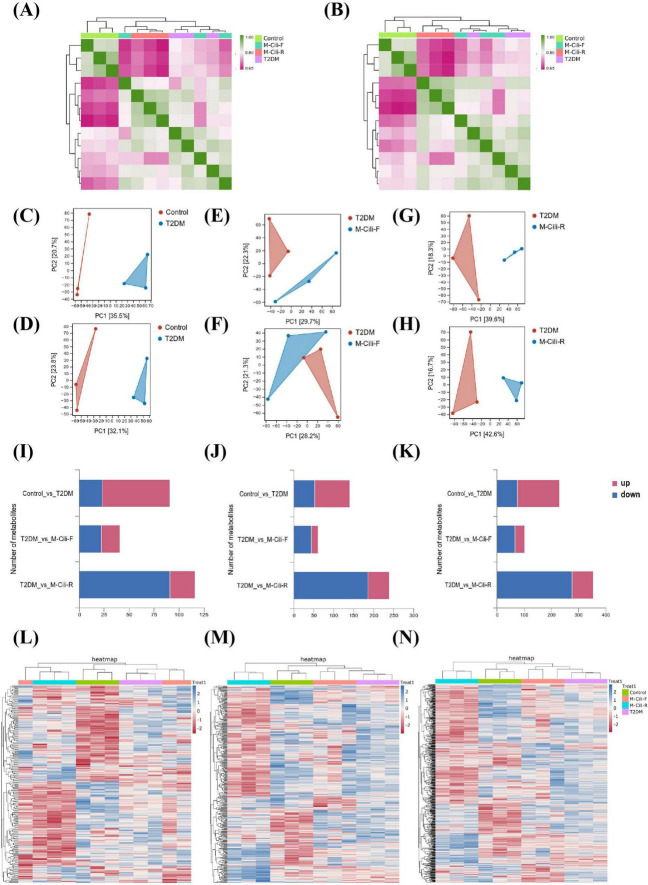
Detection of metabolites in the urine of each rat group (*n* = 3). **(A)** Sample correlation analysis in negative ion mode. **(B)** Sample correlation analysis in positive ion mode. **(C–H)** Multivariate statistical PCA, where **(C,D)** shows Control vs. T2DM, **(E,F)** show T2DM vs. M-Cili-F, and **(G,H)** show T2DM vs. M-Cili-R. **(C,E,G)** represent the negative mode, and **(D,F,H)** represent the positive mode. **(I–K)** Screening of differentially abundant metabolites, where I represents the negative mode, J represents the positive mode, and K represents the merge mode of positive and negative ions. **(L–N)** Agglomerate hierarchical clustering analysis of differentially metabolized compounds in the four groups in negative, positive and merge modes.

Non-targeted metabolomics analysis was conducted on the urine samples from each group of rats. Prior to conducting differential expression analysis, the correlations among the expression levels of substances within each group were assessed to elucidate their interrelationships (Figures 5A,B). The findings indicated that the correlations among the biological replicate samples within each group was exceptionally robust. Hierarchical clustering was subsequently employed for bidirectional clustering of both samples and metabolites, with a heatmap illustrating the variations in the abundances of identical substances across different samples (Supplementary Figures 4A,B). The PCA diagram revealed that closer proximity of sample distribution points corresponded to greater similarity in the composition and concentration of variables within those samples. This finding indicates a distinct separation between the urine metabolites from each group and those from the T2DM group (Figures 5C–H). The selection criteria for significant differentially abundant metabolites were defined as VIP > 1 and *p* < 0.05. Differentially abundant metabolites were identified in positive ion mode, negative ion mode, and merged mode; specifically, 66 downregulated and 36 upregulated metabolites were selected from the M-Cili-F group relative to the T2DM group, along with 277 downregulated and 78 upregulated metabolites identified from the M-Cili-R group compared with the T2DM group, as illustrated in Figures 5I–K.

The volcano plot depicts all the differentially abundant metabolites identified in the three comparison groups of Control vs. T2DM, T2DM vs. M-Cili-F, and T2DM vs. M-Cili-R in both positive and negative ion modes (Supplementary Figures 4C–H). The cluster analysis revealed the global disparities in metabolic profiles among the four experimental groups in negative, positive and merge modes (Figures 5L,N).

**FIGURE 6:**
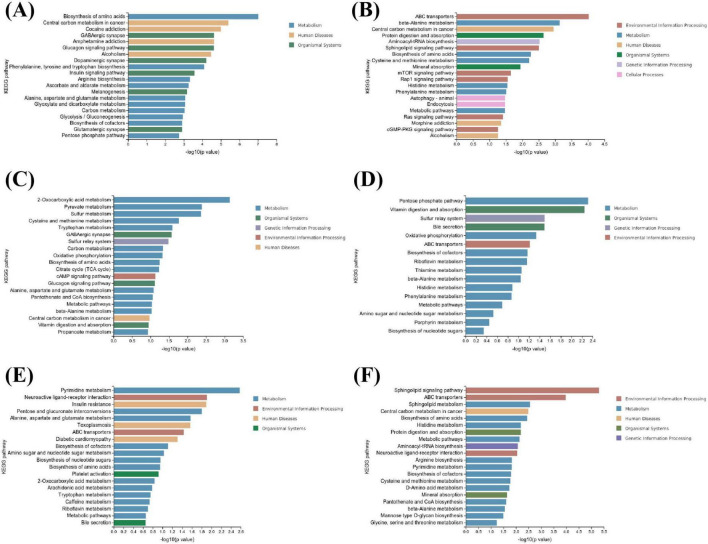
KEGG pathway analysis of differentially metabolized compounds in rat urine (*n* = 3). **(A,B)** Control vs. T2DM groups in negative mode and positive mode. **(C,D)** T2DM vs. M-Cili-F groups in negative mode and positive mode. **(E,F)** T2DM vs. M-Cili-R groups in negative mode and positive mode.

A total of 2,167 differentially abundant metabolites were identified. Among these, 230 metabolites, predominantly organic acids and derivatives (25%), organ heterocyclic compounds (14%), organic oxygen compounds (11%), benzenoids (11%), and lipids and lipid-like molecules (11%), among others, exhibited significant differences between the control and T2DM groups. There were 155 upregulated differentially abundant metabolites and 75 downregulated differentially abundant metabolites. The following KEGG enrichment pathways highlight the top 20 significantly altered signaling pathways under both the negative and positive ion modes, which primarily encompass metabolism, human diseases, organismal systems, and environmental information processing (see Figures 6A,B).

A total of 102 metabolites demonstrated significant differences in T2DM vs. M-Cili-F, primarily consisting of lipids and lipid-like molecules (23%), organic acids and their derivatives (18%), organic oxygen compounds (17%), and benzenoids (14%). Among these, 36 upregulated differentially abundant metabolites and 66 downregulated differentially abundant metabolites were detected. The top 20 significantly different signaling pathways in the negative state as well as the top 16 significantly different signaling pathways in the positive state, which mainly involve organismal systems such as vitamin digestion and absorption; genetic information processing, including sulfur relay systems; metabolic processes such as oxidative phosphorylation and bile secretion; and environmental information processing, which encompasses ABC transporters and the cAMP signaling pathway, are summarized in Figures 6C,D.

There are 355 metabolites that were significantly different between T2DM and M-Cili-R, predominantly comprising organic acids and derivatives (23%), lipids and lipid-like molecules (16%), organ heterocyclic compounds (17%), and benzenoids (10%). Among these metabolites, 78 were upregulated, and 277 were downregulated. The top 20 significantly different signaling pathways identified in the KEGG enrichment analysis for both the cationic and anionic states are depicted in Figures 6E,F. These pathways included environmental information processing pathways, such as the sphingolipid signaling pathway and ABC transporters; metabolic processes, including sphingolipid metabolism; human diseases; organismal systems, including protein digestion, absorption, and mineral absorption; and genetic information processing, such as aminoacyl-tRNA biosynthesis.

Some metabolites in the urine of the model rats after intervention with FCJ/RCJ are listed in Tables 2, 3. The results showed that rats fed FCJ excreted urine rich in anti-inflammatory substances such as chelidonic acid (CA), 8-prenylnaringenin, and phyllanthin and antidiabetic or insulin-regulating substances such as acetohexamide and L-rhamnose. Compared with those in the T2DM group, the levels of metabolites associated with NAFLD, hepatic injury, and obesity, including protoporphyrin IX, 13-keto-9Z, 11E-octadecadienoic acid, o-acetylserine, indoleacrylic acid, and glycine, were significantly lower in the M-Cili-F group. These findings imply that FCJ has potential antidiabetic and anti-inflammatory properties in rats with T2DM-associated NAFLD.

**TABLE 2:** Information on potential differentially abundant metabolites in the urine of T2DM vs. M-Cili-F rats.

No.	HMDB	Name	Adduct	KEGG	Super class	Sub class	Regulation*
1	HMDB0001520	Flavine mononucleotide	[M + H-H_2_O] +	C00061			Up
2	HMDB0014558	Acetohexamide	[M + Na] +	C06806	Organic oxygen compounds	Carbonyl compounds	Up
3	HMDB0005030	Fexofenadine	[M + H] +	C06999	Benzenoids	Diphenylmethanes	Up
4	–	2,8-quinolinediol	[M-H]-	–	Organoheterocyclic compounds	Quinolones and derivatives	Up
5	HMDB0000849	L-Rhamnose	(M-H)-	C00507	Organic oxygen compounds	Carbohydrates and carbohydrate conjugates	Up
6	HMDB0000215	N-acetyl-d-glucosamine	[M + H-CH8O4] +	C00140	Organic oxygen compounds	Carbohydrates and carbohydrate conjugates	Up
7	HMDB0062356	L-thyronine	[M + H] +	–	Organic acids and derivatives	Amino acids, peptides, and analogs	Up
8	–	5-(methylthio)salicylic acid	[M-H]-	–	Benzenoids	Benzoic acids and derivatives	Up
9	–	Phyllanthin	[M + Na] +	C10746			Up
10	–	3,5-dihydroxydecanoic acid	[M-H]-	–	Organic acids and derivatives	Medium-chain hydroxy acids and derivatives	Up
11	HMDB0012107	N-nervonoyl-d-erythro-sphingosylphosphoryl choline	[M + H] +	–	Lipids and lipid-like molecules	Phosphosphingolipids	Up
12	HMDB0006582	Lewis a trisaccharide	[M + H-H_2_O] +	–	Lipids and lipid-like molecules	Fatty acyl glycosides	Up
13	HMDB0000439	2-Furoylglycine	[M-H]-	–	Organic acids and derivatives	Amino acids, peptides, and analogs	Up
14	–	8-prenylnaringenin	[M-H]-	–	Phenylpropanoids and polyketides	Flavans	Up
15	HMDB0004089	Formylanthranilic acid	[M-H]-	C05653	Benzenoids	Benzoic acids and derivatives	Up
16	HMDB0015249	Atovaquone	[M + H] +	C06835	Benzenoids	Naphthoquinones	Up
17	–	Chelidonic acid	[M-H-CO2]-	C08476	Organoheterocyclic compounds	Pyranones and derivatives	Up
18	HMDB0004240	15-ketoprostaglandin f2 alpha	[M + H] +	–	Lipids and lipid-like molecules	Eicosanoids	Down
19	–	Lasiocarpine n-oxide	[M + H] +	–			Down
20	–	Leukotriene d4	[M + H-H_2_O] +	–	Organic acids and derivatives	Amino acids, peptides, and analogs	Down
21	HMDB0001858	4-methylphenol	[M-H]-	C01468	Benzenoids	Cresols	Down
22	HMDB0003011	O-acetylserine	[M-H]-	C00979	Organic acids and derivatives	Amino acids, peptides, and analogs	Down
23	HMDB0000241	Protoporphyrin ix	[M + H] +	C02191			Down
24	HMDB0006294	16-Hydroxypalmitic acid	(M + Na) +	C18218	Lipids and lipid-like molecules	Fatty acids and conjugates	Down
25	HMDB0003518	Homocitrate	[M-H]-	C01251	Organic acids and derivatives	Tricarboxylic acids and derivatives	Down
26	HMDB0004668	13-keto-9z,11e-octadecadienoic acid	[M + H] +	–	Lipids and lipid-like molecules	Lineolic acids and derivatives	Down
27	HMDB0000734	Indoleacrylic acid	(M + H-H_2_O) +	–	Organoheterocyclic compounds	Indoles	Down
28	HMDB0000860	(3-phenylpropionyl) glycine	[M-H]-	–	Organic acids and derivatives	Amino acids, peptides, and analogs	Down
29	–	Propaquizafop	[M + H] +	C18886	Benzenoids	2-phenoxypropionic acid esters	Down

*p < 0.05 and VIP > 1 (*n* = 3), and *p*-values are arranged in ascending order of up- and downregulation.

**TABLE 3:** Information on potential differentially abundant metabolites in the urine of T2DM vs. M-Cili-R rats.

No.	HMDB	Name	Adduct	KEGG	Super class	Sub class	Regulation*
1	HMDB0005782	Hesperetin	[M + H] +	C01709	Phenylpropanoids and polyketides	O-methylated flavonoids	Up
2	HMDB0015335	Mitoxantrone	[M-H]-	C11195	Benzenoids	Anthraquinones	Up
3	HMDB0000328	12-ketodeoxycholic acid	[M + H] +	–	Lipids and lipid-like molecules	Bile acids, alcohols and derivatives	Up
4	HMDB0000415	beta-muricholic acid	[M + H-2H_2_O] +	C17726	Lipids and lipid-like molecules	Bile acids, alcohols and derivatives	Up
5	HMDB0004951	N-(eicosanoyl) sphingosine (Cer)	[M + H] +	–	Lipids and lipid-like molecules	Ceramides	Up
6	HMDB0030282	Cinchonine	[M-H]-	C06528	–	–	Up
7	HMDB0002536	Isodeoxycholic acid	[M-H-H_2_O]-	C17661	Lipids and lipid-like molecules	Bile acids, alcohols and derivatives	Up
8	HMDB0034227	Alpha-tochopheryl acetate	[M + NH4] +	–	Lipids and lipid-like molecules	Quinone and hydroquinone lipids	Up
9	HMDB0014558	Acetohexamide	[M + Na] +	C06806	Organic oxygen compounds	Carbonyl compounds	Up
10	HMDB0014558	Acetohexamide	[M + Na] +	C06806	Organic oxygen compounds	Carbonyl compounds	Up
11		3-dehydrocholic acid	[M-H]-	–	–	–	Up
12	HMDB0003153	Epigallocatechin gallate	[M + H] +	C09731	Phenylpropanoids and polyketides	Flavans	Up
13	HMDB0001238	N-Acetylserotonin	[M-H]-	C00978	Organoheterocyclic compounds	Hydroxyindoles	Up
14	HMDB0000269	Pro-Trp	[M + H] +	C00836	Organic nitrogen compounds	Amines	Up
15	HMDB0012252	Linoleoyl ethanolamide	[M + H] +	–	Organic nitrogen compounds	Amines	Up
16	HMDB0029540	Nobiletin	[M + Na] +	C10112	Phenylpropanoids and polyketides	O-methylated flavonoids	Up
17	HMDB0000852	beta-sitosterol	[M + H-H_2_O] +	C01753	Lipids and lipid-like molecules	Stigmastanes and derivatives	Up
18	HMDB0002183	Docosahexaenoic acid	[M-H]-	C06429	Lipids and lipid-like molecules	Fatty acids and conjugates	Up
19	HMDB0000619	Cholic acid	[M + NH4] +	C00695	Lipids and lipid-like molecules	Bile acids, alcohols and derivatives	Up
20	HMDB0006961	Hydroxyacetone	(M + CH3COO)-	C05235	Organic oxygen compounds	Carbonyl compounds	Down
21		Octanoylcarnitine	[M + H] +	–	Lipids and lipid-like molecules	Fatty acid esters	Down
22	HMDB0000001	1-methyl-l-histidine	[M + H-CH_2_O_2_] +	C01152	Organic acids and derivatives	Amino acids, peptides, and analogs	Down
23	HMDB0000290	Udp-n-acetylglucosamine	[M-H]-	C00043	Nucleosides, nucleotides, and analogs	Pyrimidine nucleotide sugars	Down
24	HMDB0004063	Metanephrine	[M + H-2H_2_O] +	C05588	Benzenoids	Methoxyphenols	Down
25		L-glutarylcarnitine	[M + H] +	–	Lipids and lipid-like molecules	Fatty acid esters	Down
26	HMDB0000277	Sphingosine 1-phosphate	[M + H] +	C06124	Lipids and lipid-like molecules	Phosphosphingolipids	Down
27	HMDB0006029	N-acetylglutamine	[M-H]-	–	Organic acids and derivatives	Amino acids, peptides, and analogs	Down
28		Isobutyryl-l-carnitine	[M + H] +	–	Lipids and lipid-like molecules	Fatty acid esters	Down
29		Hexanoyl-l-carnitine	[M + H] +	–	Lipids and lipid-like molecules	Fatty acid esters	Down
30		3-hydroxybutyryl carnitine	[M + H] +	–	Lipids and lipid-like molecules	Fatty acid esters	Down
31	HMDB0000842	Quinaldic acid	(M + H) +	C06325	Organoheterocyclic compounds	Quinoline carboxylic acids	Down
32	HMDB0240212	Dimethylguanidino valeric acid	[M + H] +	–	Organic acids and derivatives	Short-chain keto acids and derivatives	Down
33	HMDB0003269	Nicotinuric acid	[2M-H]-	C05380	Organic acids and derivatives	Amino acids, peptides, and analogs	Down
34	HMDB0000564	PC (16:0/16:0)	[M + H] +	C00157	Lipids and lipid-like molecules	Glycerophospho cholines	Down
35	HMDB0000085	Deoxyguanosine	[M-H]-	C00330	Nucleosides, nucleotides, and analogs	Purine 2’-deoxyribonucleosides	Down
36	HMDB0002107	1,2-benzenedicarboxylic acid	[M + H-H_2_O] +	C01606	Benzenoids	Benzoic acids and derivatives	Down
37	HMDB0001209	Allantoic acid	[M-H]-	C00499	Organic acids and derivatives	Amino acids, peptides, and analogs	Down
38	HMDB0012101	N-oleoyl-d-erythro-sphingosylphosphoryl choline	[M + H] +	–	Lipids and lipid-like molecules	Phosphosphingolipids	Down
39	HMDB0000191	L-Aspartate	(M + H) +	C00049	Organic acids and derivatives	Amino acids, peptides, and analogs	Down
40	HMDB0010163	2-oleoyl-1-stearoyl-sn-glycero-3-phosphoserine	[M-H]-	–	Lipids and lipid-like molecules	Glycerophosphoserines	Down
41	HMDB0000678	Isovalerylglycine	[M-H]-	–	Organic acids and derivatives	Amino acids, peptides, and analogs	Down
42	HMDB0001866	3,4-dihydroxymandelic acid	[M-H-H_2_O]-	C05580	Benzenoids	Benzenediols	Down
43	HMDB0000562	Creatinine	[M-H]-	C00791	Organic acids and derivatives	Amino acids, peptides, and analogs	Down
44	HMDB0000875	Trigonelline	[M + H] +	C01004	Alkaloids and derivatives	–	Down
45	HMDB0000187 HMDB0062263	L-Serine	(M + CH3CN + H) +	C00065	Organic acids and derivatives	Amino acids, peptides, and analogs	Down

*p < 0.05 and VIP > 1 (*n* = 3), and p values are arranged in ascending order of up- and downregulation.

Rats fed RCJ excreted urine with increased levels of bile acids to regulate cholesterol homeostasis. Moreover, the active compounds that modulate cholesterol and blood lipid levels, including hesperetin, epigallocatechin gallate, and nobiletin, were significantly upregulated in the M-CILI-R group compared with those in the T2DM group, confirming the pronounced hypolipidemic and hypoglycemic effects of RCJ. Conversely, RCJ significantly decreased the levels of various acylcarnitines, which are critical biomarkers linked to metabolic disorders and diabetes. Additionally, RCJ decreased the concentrations of specific amino acids, such as L-aspartate and L-serine, in the urine. These findings indicate that RCJ has the potential to ameliorate the abnormal metabolism of fatty acids and amino acids in rats with T2DM-associated NAFLD.

### 3.7 FCJ/RCJ improves aberrant gene expression in rats with T2DM-associated NAFLD

**FIGURE 7:**
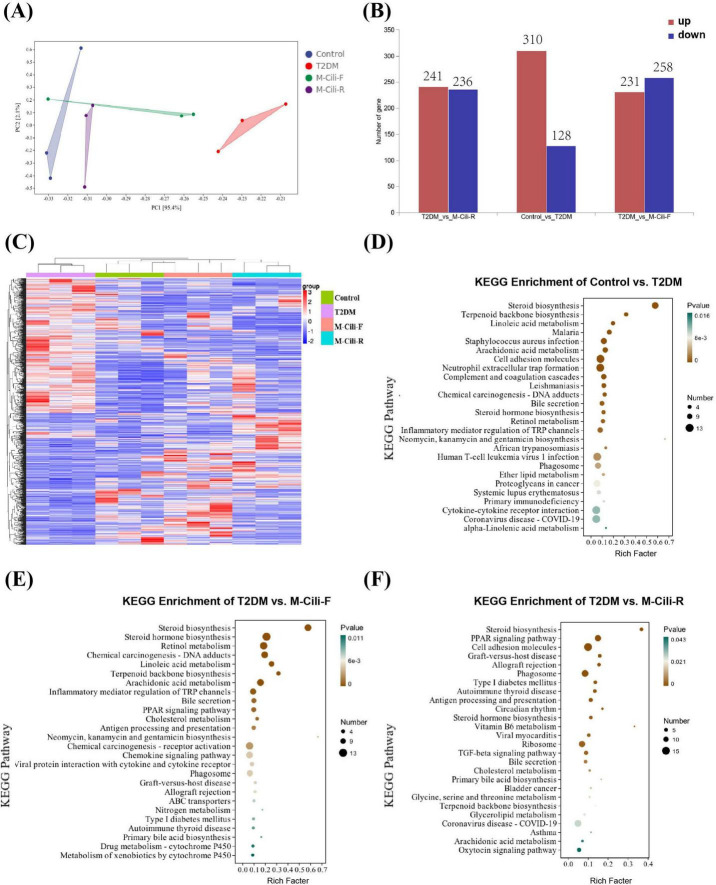
DEG analysis of different groups of rats (*n* = 3). **(A)** PCA of 12 samples in each group. **(B)** The number of DEGs in different groups with *p* < 0.05. **(C)** Heatmap of DEGs in different groups. Red indicates upregulated genes, blue indicates downregulated genes, and gray indicates no difference in genes. **(D–F)** KEGG enrichment analysis of DEGs in different treatment groups. **(D)** Control vs. T2DM. **(E)** T2DM vs. M-Cili-F. **(F)** T2DM vs. M-Cili-R (*p* < 0.05).

RNA sequencing (RNA-seq) was performed to detect the hepatic transcription levels in the rats. PCA revealed that the T2DM group was separated from the control, M-Cili-F and M-Cili-R groups (Figure 7A). Compared with the control group, the T2DM group presented 310 upregulated genes and 128 downregulated genes. Compared with the T2DM group, the M-Cili-F group had 231 upregulated genes and 258 downregulated genes, and the M-Cili-R group had 241 upregulated genes and 236 downregulated genes (Figure 7B). Volcano plots were constructed to show the DEGs in the different groups compared with those in the T2DM group (Supplementary Figures 4I–K). In a heatmap that was constructed to summarize these differences, the DEGs are represented horizontally, and both FCJ and RCJ restored the expression of several genes in the T2DM-associated NAFLD rats to near the levels in the control group (Figure 7C).

To characterize the functional consequences of the DEGs, KEGG pathway analyses were performed. A comparison of the control and T2DM groups revealed that 44 KEGG terms were significantly enriched (*p* < 0.05), and Figure 7D shows the top 26 terms. Compared with the T2DM group, the M-Cili-F group had 43 KEGG terms that were significantly enriched (*p* < 0.05). Figure 7E shows the top 26 terms, most of which were related to metabolism, human diseases, organismal systems, and environmental information processing, while the M-Cili-R group had 26 KEGG terms that were significantly enriched (*p* < 0.05). Figure 7F shows the 26 terms. The KEGG results revealed that the sterol metabolism pathway was significantly enriched in each of the three comparisons, but the DEGs of the groups treated with different kinds of Cili juices were enriched in different pathways. The DEGs of the M-Cili-F group were mostly enriched in steroid biosynthesis, steroid hormone biosynthesis, retinol metabolism, linoleic acid metabolism, terpenoid backbone biosynthesis and inflammatory mediator regulation, whereas those of the M-Cili-R group were enriched in steroid biosynthesis, the Peroxisome proliferator-activated receptor (PPAR) signaling pathway, cell adhesion molecules, the TGF-beta signaling pathway and bile secretion. Both FCJ/RCJ-treated group DEGs were more likely to be enriched in lipid metabolism and the immune and endocrine systems.

### 3.8 qRT–PCR and western blot analysis to validate the selected genes

**FIGURE 8:**
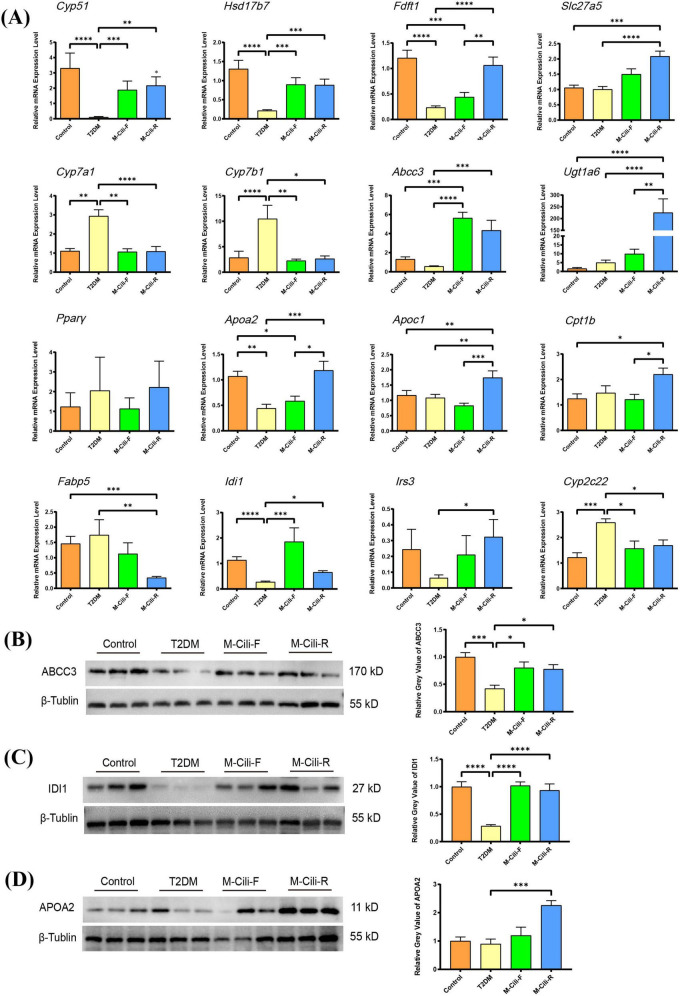
Expression analyses of transcription or protein levels of selected genes. **(A)** Expression analyses of selected genes by qRT–PCR. The data represent the means ± SDs from six biological replicates with three technical replicates. **(B–D)** FCJ/RCJ upregulated ABCC3, IDI1, and APOA2 expression in the liver. FCJ and RCJ significantly upregulated hepatic ABCC3 **(B)** and IDI1 **(C)** expression, and RCJ significantly upregulated hepatic APOA2 **(D)** expression in T2DM rats. The data represent the means ± SDs from three biological replicates with three technical replicates. *p < 0.05, ***p < 0.001, ****p < 0.0001.

To confirm the credibility of the RNA-seq analysis, qRT-PCR was performed to validate the expression levels of DEGs in hepatic tissues. Based on the fold change and KEGG enrichment analysis results, we selected the genes cytochrome P450, family 51 (*Cyp51*), 17β-hydroxysteroid dehydrogenase (*Hsd17b7*), farnesyl diphosphate farnesyl transferase 1 (*Fdft1*), solute carrier family 27 member 5 (*Slc27a5*), which are related to sterol metabolism, cytochrome P450 family 7 subfamily A member 1 (*Cyp7a1*), cytochrome P450 family 7 subfamily B member 1 (*Cyp7b1*), ATP binding cassette subfamily C member 3 (*Abcc3*) and UDP glucuronosyltransferase family 1 member A6 (*Ugt1a6*), which are related to bile acid metabolism and ABC transporters, to detect transcript levels using qRT-PCR. Additionally, peroxisome proliferator-activated receptor γ (*PPAR*γ), apolipoprotein A2 (*Apoa2*), apolipoprotein C1 (*Apoc1*), carnitine palmitoyl transferase 1B (*Cpt1b*), fatty acid binding protein 5 (*Fabp5*) (related to the PPAR signaling pathway), insulin receptor substrate 3 (*Irs3*) (related to T2DM), and isopentenyl-diphosphate delta isomerase 1 (*Idi1*) cytochrome P450, family 2, subfamily c, polypeptide 22 (*Cyp2c22*) (related to terpenoid biosynthesis and retinol metabolism) were analyzed, as shown in Figure 8A.

Compared with those in the control group, the expression levels of *Cyp51, Hsd17b7, Faft1, Apoa2*, and *Idi1* were significantly downregulated, whereas the expression levels of *Cyp7a1, Cyp7b1* and *Cyp2c22* were upregulated in the T2DM group (*p* < 0.05). Other genes, such as *Slc27a5, Ugt1a6, Abcc3, Apoc1, Cpt1b*, and *Irs3*, were not significantly different between the control and T2DM groups but were recovered in the M-Cili-F group or M-Cili-R group (*p* < 0.05). Compared with those in the T2DM group, the expression levels of *Cyp51, Hsd17b7, Cyp7a1, Cyp7b1, Abcc3, Idi1*, and *Cyp2c22* in the M-Cili-F group were significantly greater (*p* < 0.05), and those of *Cyp51, Hsd17b7, Fdft1, Slc27a5, Cyp7a1, Cyp7b1, Abcc3, Ugt1a6, Apoa2, Apoc1, Fabp5, Irs3, Idi1*, and *Cyp2c22* in the M-Cili-R group were restored compared with those in the T2DM group (*p* < 0.05). *Cyp51* and *Fdft1* are essential enzymes for sterol biosynthesis, and *Hsd17b7* is involved in the cholesterol synthesis pathway. *Slc27a5* plays a tissue-specific role in regulating bile acid synthesis and reducing lipid accumulation. Regulating the levels of *Cyp7a1, Cyp7b1, Abcc3*, and *Ugt1a6* could maintain bile acid homeostasis through enterohepatic circulation to protect the liver. *Apoa2* and *Apoc1* play important roles in cholesterol metabolism by increasing the level of cholesterol efflux, *Cpt1b* is a rate-limiting enzyme in fatty acid β-oxidation, and *Idi1* plays crucial roles in increasing the production of terpenoids to protect the liver through anti-inflammatory and antioxidation effects. These findings demonstrated that FCJ/RCJ regulated hepatic sterol synthesis, facilitated cholesterol efflux, suppressed fatty acid synthesis, enhanced fatty acid β-oxidation, maintained bile acid homeostasis, and promoted the production of anti-inflammatory substances in rats with T2DM-associated NAFLD.

Moreover, *Fdft1, Apoa2, Apoc1, Cpt1b*, and *Ugt1a6* expression levels significantly differed between the two groups treated with FCJ and RCJ (*p* < 0.05). M-Cili-R was superior to M-Cili-F in increasing the transcription levels of *Cpt1b, Apoa2, Apoc1*, and *Ugt1a6* in T2DM rats, indicating that RCJ was more effective than fermented juice in promoting lipid consumption and cholesterol efflux and regulating bile acid metabolism in T2DM-associated NAFLD rats. The obtained qRT–PCR results further confirmed the reliability of the transcriptome sequencing findings.

Furthermore, a Western blot analysis was performed to validate the protein abundances of ABCC3, IDI1, and APOA2 in the liver tissues of the rats in each group. As depicted in Figures 8B–D, the expression levels of the ABCC3 and IDI1 proteins were markedly decreased in the T2DM group and significantly decreased after FCJ/RCJ intervention (*p* < 0.05), whereas the expression of the APOA2 protein was significantly increased after RCJ intervention (*p* < 0.05). These findings further suggest that FCJ/RCJ play pivotal roles in maintaining lipid homeostasis and that the cholesterol efflux effect of RCJ is clearly more pronounced.

### 3.9 Joint analysis of FCJ/RCJ component content and the liver transcriptome

**FIGURE 9:**
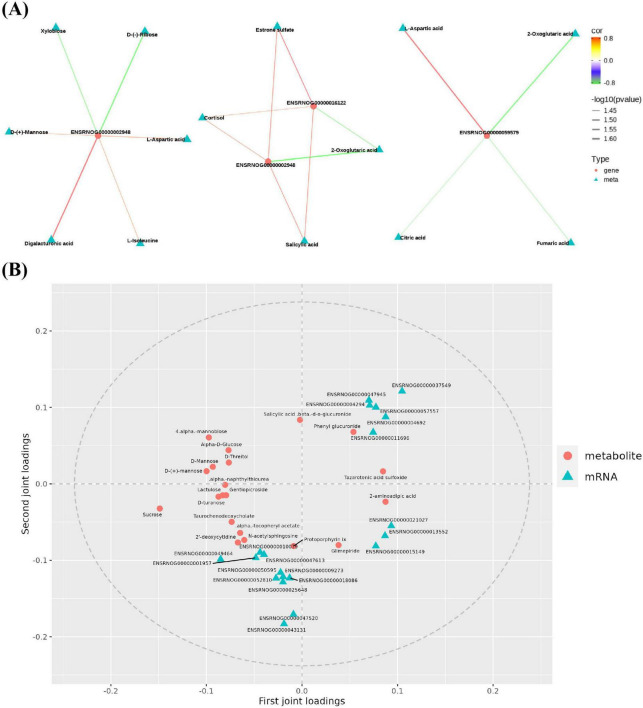
Joint analysis of mass spectrometry results and sequencing results. **(A)** Analysis diagrams of the joint pathway networks between the significantly different components of FCJ/RCJ and the DEGs of the hepatic transcriptome. The red dots represent the differential genes, whereas the blue–green triangles represent the differential components of FCJ/RCJ. Red lines indicate positive correlations, and green lines indicate negative correlations. The Pearson correlation coefficients exceeded 0.8. **(B)** Enhanced integration of metabolomics and transcriptomics for integrated analysis.

To obtain the common pathway information of the mapping results of differential genes and components, the differential components in FCJ/RCJ and DEGs in transcriptomics were simultaneously mapped to the KEGG pathway database. The correlation network diagrams were subsequently constructed for the genes and metabolites within the shared pathway, with a focus on those exhibiting a Pearson correlation coefficient exceeding 0.8, which culminated in the integrated analysis results (Figure 9A). *Abcc3* (ENSRNOG00000002948) was significantly positively correlated with digalacturonic acid, L-isoleucine, D-mannose, and L-aspartic acid and significantly negatively correlated with xylobiose and D-ribose. Moreover, *Abcc3* and 3-hydroxy-3-methylglutaryl-CoA synthase 1 (*Hmgcs1*) (ENSRNOG00000016122) were significantly positively correlated with components such as cortisol, ester sulfate, and salicylic acid in FCJ/RCJ and significantly negatively correlated with 2-oxoglutaric acid. Glutamic-pyruvic transaminase 2 In the joint analysis (*Gpt2*) (ENSRNOG00000059579) was significantly positively correlated with L-aspartic acid and significantly negatively correlated with 2-oxoglutaric acid, citric acid, and fumaric acid. *Gpt2* was downregulated in M-Cili-R compared with T2DM group.

### 3.10 Enhanced integration of metabolomics and transcriptomics for integrated analysis

All genes and metabolites were selected to establish the O2PLS model, thereby identifying the significant variables that impact other omics data. Figure 9B shows the load distribution of the transcriptome and metabolite associations. The dots in the figure symbolize genes or metabolites, where a higher absolute value in the coordinates indicates a stronger association between this element and other omics data. The results revealed that some DEGs, including stearoyl-CoA desaturase (*Scd*), cytochrome P450, family 2, subfamily c, polypeptide 13 (*Cyp2c13*), cytochrome P450, family 2, subfamily c, polypeptide 11 (*Cyp2c11*), solute carrier family 22 member 8 (*Slc22a8*), prolactin receptor (*Prlr*), and sulfotransferase family 1E member 1 (*Sult1e1*) genes, were the critical variables that influence urine metabolites, and some metabolites, such as Protoporphyrin IX (Proto IX), alpha-tocopheryl acetate, taurochenodeoxycholate, and D-turanose, also influenced the results of hepatic transcriptomics. *Scd* participates in fatty acid synthesis, and its transcriptional level is increased in T2DM rats and can be repressed by FCJ/RCJ. *Cyp2c13* expression is increased during liver injury, and the increase in *Cyp2c13* expression in T2DM-associated NAFLD rats was clearly inhibited by FCJ in this study. *Cyp2c11* is correlated with arachidonic acid metabolism, and terpenoid compounds can suppress *Cyp2c13* expression, which is in accordance with the outcomes of this study. The upregulated expression of *Slc22a8* can indirectly affect bile acid secretion, and both FCJ and RCJ significantly diminished the upregulated *Slc22a8* expression in T2DM-associated NAFLD. *Prlr* has a regulatory effect on liver insulin sensitivity, and its expression is lower in T2DM. This study revealed that FCJ/RCJ can strongly restore the expression level of *Prlr*. *Sult1e1* actively participates in the catabolic process of estrogen and interacts with *PPAR*γ, contributing to the pathogenesis of atherosclerosis. In this study, the augmented expression of *Sult1e1* in T2DM-associated NAFLD was clearly repressed by FCJ. The abnormal accumulation of Proto IX within the liver can induce cellular apoptosis and inflammation, potentially culminating in liver failure and bile stasis. In this study, both FCJ and RCJ alleviated the excessive accumulation of Proto IX in T2DM-associated NAFLD rats. Therefore, some genes or metabolites related to lipid metabolism, bile acid metabolism, insulin sensitivity, inflammation, and liver injury may have a substantial impact on the outcomes of other omics analyses.

## 4 Discussion

T2DM involves metabolic dysfunction of multiple systems, such as significant hyperglycemia, hyperlipidemia, relative insulin resistance, and liver function damage. Research has revealed that numerous medicinal plants are extensively employed in the treatment of diabetes (34), inflammation (35), cardiovascular diseases (36), and other disorders. For example, flavonoid compounds have demonstrated considerable efficacy in alleviating diabetes (37). Dietary polyphenols have antidiabetic potential, including reduce blood glucose levels, oxidative stress, and improve IR (38). Cili is rich in flavonoids, polyphenols, and various vitamins, among other beneficial components. However, no comparative analysis has been conducted on the alterations in the composition and concentration of Cili juice before and after fermentation or on the differences and similarities in its efficacy in preventing and treating T2DM-associated NAFLD. Therefore, the aim of this research was to assess the beneficial effects of fermented Cili juice and raw Cili juice in ameliorating T2DM-associated NAFLD.

The influence of fermentation on small molecule components, particularly flavonoids and polyphenols, warrants further investigation. Recent study has demonstrated that fermentation can substantially alter the bioavailability and bioactivity of these compounds via structural modifications (39). Consequently, UPLC/MS was employed for semi-quantitative analysis of small molecule compounds in FCJ and RCJ in this study. According to the results of the analysis of FCJ with UPLC/MS, the contents of 32 flavonoids, polyphenols, and vitamins, including fisetin, luteolin, tyrosol, and pyridoxine, increased in FCJ. Fisetin and luteolin primarily exhibit anti-inflammatory, antiviral and antioxidant properties, tyrosol exert antioxidant and pancreatic protective properties, and pyridoxine also exhibit high anti-Inflammatory and antibacterial activities (40–45). The substantial increase in small-molecule flavonoids, polyphenolic and vitamins compounds in FCJ enhances the overall anti-inflammatory efficacy of FCJ, making it more pronounced. Among the downregulated components in FCJ, 42 flavonoids, vitamins, and polyphenols, such as epicatechin, capsaicin, eriodyctiol, and nicotinic acid, were reduced. These substances mainly serve to lower lipid levels and protect the liver (46–48). Among the substances that were not significantly different between FCJ and RCJ, 46 distinct flavonoids, vitamins, and polyphenolic compounds were identified, such as quercetin, ascorbic acid, and rhamnetin, which possess potent antidiabetic, anti-inflammatory, and antioxidant properties (49–52). Due to methodological constraints of non-targeted metabolomics, a more in-depth analysis of the polysaccharide (a high-molecular-weight polymer) in FCJ/RCJ could not be conducted. However, it is undeniable that polysaccharides extracted from Cili have demonstrated certain anti-diabetic effects (10, 11, 13). In summary, both FCJ and RCJ contain a diverse array of antidiabetic substances capable of regulating glucose and lipid metabolism, with anti-inflammatory substances being more prevalent in FCJ and lipid-lowering substances being more abundant in RCJ.

After 20 weeks of intervention in T2DM-associated NAFLD rats, we found that FCJ and RCJ reduced the levels of FBG, TG, TC, LDL-C, AST, and ALT in the serum of T2DM rats with liver injury, and the accumulation of lipid droplets in hepatocytes was significantly improved in the tissue sections according to the staining results. In addition, both Cili juices improved insulin secretion by islet β-cells in T2DM-associated NAFLD rats. Therefore, the oral administration of FCJ and RCJ could protect liver and pancreas tissue, reduce lipid metabolism disorders and promote glucose metabolism in T2DM-associated NAFLD rats.

Following the comparison of differentially metabolized substances in the urine of rats across groups post-FCJ/RCJ intervention, a series of metabolites with notable alterations were identified. The differences in the effects of FCJ and RCJ on T2DM-associated NAFLD rats were elucidated with analyzing urinary metabolite profiles. The findings indicated that urinary flavin mononucleotide (FMN), fexofenadine, phyllanthin, Lewis Atrisaccharide, and urinary FMN levels were markedly elevated in FCJ-treated rats compared with those in T2DM group. Additionally, the levels of metabolites such as 2-furoylglycine and CA were significantly increased. FMN possesses anti-inflammatory properties, whereas fexofenadine effectively inhibits inflammation and histamine release in patients with diabetic nephropathy (53, 54). Phyllanthin mitigates oxidative stress and inflammation, and Lewis Atrisaccharide prevents inflammation-induced metabolic disorders (55, 56). Both 2-furoylglycine and CA exhibit anti-inflammatory effects (57, 58). The observed increases in multiple anti-inflammatory substances substantiates that FCJ intake significantly enhances the body’s anti-inflammatory capacity. Furthermore, the urinary excretion of acetohexamide, L-thyronine, formylanthranilic acid, atovaquone, and 5-(methylthio) salicylic acid notably increased following FCJ intervention. Acetohexamide is an antidiabetic sulfonamide medication (59). A low level of L-thyronine may pose a potential risk for type 2 diabetic peripheral neuropathy (60). Formylanthranilic acid plays a role in tryptophan metabolism and may serve as a potential prognostic marker for diabetic nephropathy (61). Atovaquone inhibits the proliferation of hepatocellular carcinoma cells, and 5-(methylthio)salicylic acid is a beneficial natural phenolic compound (62). The significant increases in these metabolites in the urine of FCJ-treated rats further corroborate the potential of FCJ in mitigating diabetic complications and providing hepatic protection. In addition to markedly decreasing the levels of urinary inflammatory metabolites, such as 15-ketoprostaglandin F2α and leukotriene D4, FCJ also reduced lipid levels in T2DM-associated NAFLD rats (63, 64). Research has indicated that the concentration of indoleacrylic acid is elevated in the urine of obese mice, and that LTD4 promotes lipid accumulation (65). In this study, both indoleacrylic acid and LTD4 levels were reduced following FCJ intervention. Furthermore, several potential markers of liver injury or diabetes were also notably diminished after FCJ treatment. Abnormal accumulation of Proto IX in the liver can result in hepatocyte damage, inflammation, and potentially liver failure and cholestasis (66, 67). 16-Hydroxypalmitic acid has been shown to be positively correlated with the diagnosis of diabetic cardiovascular autonomic neuropathy (68). This study revealed that FCJ significantly lowered urinary homocitrate levels, and multiple studies have suggested that plasma citrate levels may be positively associated with various complications of T2DM (69, 70). (3)-Phenylpropionyl glycine, an acyl glycine, has been found to increase urinary excretion in patients with T2DM, serving as a potential marker for T2DM (71). These findings highlight the substantial antidiabetic, anti-inflammatory, and lipid homeostasis benefits of FCJ.

In the present study, the levels of epigallocatechin gallate (EGCG), nobiletin, and docosahexaenoic acid in the urine of RCJ-treated M-Cili-R rats were significantly greater than those in the T2DM group. Additionally, a significant increase in acetohexamide was noted. Research has demonstrated that EGCG possesses antihyperglycemic and antilipemic properties, whereas nobiletin inhibits hepatic lipogenesis and insulin resistance and promotes fatty acid oxidation (72–74). Docosahexaenoic acid has antidiabetic effects (75). These findings highlight the potent lipid-lowering and antidiabetic capabilities of RCJ. Additionally, the concentrations of alpha-tocopheryl acetate, N-acetylserotonin, and linoleoyl ethanolamide, which possess antioxidant and anti-inflammatory properties, were significantly elevated following RCJ treatment (76). β-Sitosterol, a dietary phytosterol, may play a role in cholesterol metabolism or exert an anti-inflammatory effect, whereas hesperetin has been shown to lower cholesterol levels. The expression levels of β-sitosterol and hesperetin were significantly greater in the M-Cili-R group than in the T2DM group. Bile acids, derived from cholesterol catabolism, maintain bile acid homeostasis through the enterohepatic circulation. Excess bile acids are excreted to preserve systemic homeostasis (77). Our study revealed that RCJ intake significantly enhanced the urinary excretion of various bile acids in rats with T2DM-associated NAFLD, thereby maintaining bile acid homeostasis and suggesting that compared with FCJ, RCJ has a greater capacity to regulate cholesterol homeostasis. Furthermore, RCJ has notable antidiabetic, lipid-lowering, and antioxidant activities.

Moreover, we observed significant reductions in several differentially abundant metabolites linked to the development of diabetes in the urine of rats subjected to RCJ intervention. UDP-N-acetylglucosamine functions as a glucose sensor, and the concentration of allantoic acid is positively correlated with blood glucose levels (78). Isobutyl-L-carnitine levels are elevated in the plasma of patients with gestational diabetes patients, 3-hydroxybutyrylcarnitine is increased in the plasma of individuals with T2DM, and excessive nicotinic acid may induce oxidative stress and insulin resistance, thereby increasing the risk of T2DM (79). Trigonelline levels in urine are associated with glucose metabolism in individuals with T2DM and obesity, and hydroxyacetone is related to the progression of diabetes complications (80). In this study, all the aforementioned potential biomarkers of diabetes were markedly reduced following RCJ intervention. Dimethylguanidino valeric acid serves as an independent plasma biomarker for NAFLD, predicting the onset of T2DM and demonstrating a positive correlation with abnormally elevated lipid levels (81). The upregulated expression of N-acetylglutamine disrupts normal amino acid metabolism in the liver (82). L-pipecolic acid was also significantly elevated in the serum of patients with chronic liver disease and metabolic syndrome accompanied by hepatocellular cancer (83). The amelioration of these abnormal indicators in this study further suggests that RCJ exerts a substantial effect on T2DM complicated with liver injury. Additionally, RCJ also decreased the levels of inflammatory markers, including quinaldic acid, metanephrine, sphingosine 1-phosphate, and PC (16:0/16:0), in the urine of T2DM rats, which indicated that RCJ exerts an anti-inflammatory effect (84). The alterations in the concentrations of these metabolites reaffirmed that RCJ plays a crucial role in regulating glycolipid metabolic disorders, managing diabetes, protecting the liver, and mitigating inflammation in T2DM-associated NAFLD.

In conclusion, the compositional disparities between FCJ and RCJ lead to substantial variations in their therapeutic effects on T2DM-associated NAFLD, especially with regard to anti-inflammatory and steroid homeostasis regulation properties. The aforementioned urine metabolomics findings demonstrate that FCJ primarily exerts its effects through anti-inflammatory, hypoglycemic, and lipid-lowering mechanisms, whereas RCJ is more likely to function by modulating bile acid metabolism, mitochondrial oxidation, and blood lipid and glucose levels.

Intervention with FCJ/RCJ can influence the transcriptome in certain metabolic diseases. In our study, we found that FCJ/RCJ affected the biosynthesis of steroids and steroid hormones, cholesterol metabolism, bile secretion and bile acid homeostasis, the PPAR signaling pathway and other pathways in T2DM rats. After FCJ/RCJ treatment, the rats tended to have more active metabolism, and their metrics were similar to those of the control group. The results of the KEGG analysis of the RNA-seq data revealed that the DEGs of the FCJ/RCJ-treated groups were most enriched in cholesterol metabolism, bile secretion and the PPAR pathway. To maintain cholesterol homeostasis, the body further coordinates a series of molecular pathways for the synthesis, uptake, storage, and efflux of cholesterol (85). Cholesterol as serves not only a critical structural component of animal cell membranes but also a precursor for the biosynthesis of steroid hormones, bile acids, and fat-soluble vitamins (86). Consequently, the maintenance of cholesterol homeostasis is rigorously controlled through both biosynthetic and absorptive mechanisms. Bile acids facilitate the intestinal absorption of lipids, increase insulin sensitivity, and regulate systemic lipid metabolism. The enterohepatic circulation of bile acids ensures the dynamic equilibrium of bile acid homeostasis and prevents excessive bile secretion, which could otherwise lead to liver damage. In our study, we observed significant reductions in the transcript levels of *Cyp51* and *Fdft1*, which are essential enzymes for sterol biosynthesis, in the T2DM group compared with those in the control group. These levels were restored following intervention with FCJ/RCJ. Additionally, the expression pattern of *Hsd17b7*, a gene related to steroid hormone synthesis, followed a similar trend. The key observation is that current research demonstrates a pathophysiological mechanism in T2DM-associated NAFLD wherein IR induces lipid metabolism disorders. This subsequently upregulates enzymes associated with cholesterol synthesis, leading to the accumulation of cholesterol and its metabolites (87). Such accumulation activates inflammatory and adipogenic pathways, thereby exacerbating liver damage. However, this study reveals that in T2DM-associated NAFLD rats maintained on a HFD for 34 weeks, significant hepatic steatosis, impaired liver function, and elevated serum TG level were observed, but the gene expressions of the indicators involved in steroid synthesis exhibited a significant decrease compared to the control group (*p* < 0.05). In contrast, FCJ/RCJ-intervened groups significantly restored the expression of sterol synthesis-related enzymes to normal levels (*p* < 0.05). Based on existing literature (88), we hypothesize that prolonged lipotoxicity may severely impair hepatocyte function, while chronic high cholesterol exposure could induce mitochondrial dysfunction and oxidative stress in hepatocytes, ultimately resulting in hepatocyte failure. This failure likely disrupts the functionality of certain sterol synthesis enzymes, causing sterol imbalance in the body and forming a complex feedback loop. Furthermore, long-term FCJ/RCJ intervention in T2DM-associated NAFLD rats appears to protect hepatocyte function and preserve sterol homeostasis. Simultaneously, excessive cholesterol accumulation in the liver continues to disrupt steroid hormone synthesis and bile acid homeostasis in T2DM-associated NAFLD rats. Regulating the expression of the bile acid synthesis enzymes CYP7A1 and CYP7B1 is the main compensatory mechanism to prevent liver injury caused by excessive bile secretion. Multidrug resistance protein 3 (*Mrp3*, also known as *Abcc3*) prevents the excessive accumulation of anionic substrates in hepatocytes, exerting a compensatory and protective effect. *Slc27a5* plays a tissue-specific role in regulating bile acid synthesis and reducing lipid accumulation (89). In our study, the increase in ALT in the T2DM group was accompanied by significantly increased hepatic mRNA expression levels of *Cyp7a1* and *Cyp7b1* (*p* < 0.05), and the expression level of *Cyp7b1* was greater than that of *Cyp7a1*, confirming that bile acid metabolism is enhanced in diabetic rats. Studies have shown that the levels of bile acids and ALT are elevated in patients with T2DM-associated NAFLD and that the levels of secondary bile acids can increase in patients with NAFLD (90, 91). In our research, the levels of *Cyp7a1* and *Cyp7b1* in the liver were significantly lower than those in the T2DM group, and the level of *Abcc3* was significantly greater in the FCJ/RCJ intervention groups than in the T2DM group (*p* < 0.001), which resulted in the removal of excessive bile acids to protect the liver and maintain bile acid synthesis balance through enterohepatic circulation. FCJ intervention significantly increased *Slc27a5* expression in T2DM rats (*p* < 0.05), especially RCJ (*p* < 0.001), reducing liver lipid accumulation. In addition, in our study, compared with T2DM, RCJ significantly increased the mRNA expression level of *Ugt1a6* (*p* < 0.001). Related studies have confirmed that the expression of *Ugt1a6* is upregulated during the process of increasing bile acid metabolism and inhibiting bile acid synthesis, which may be related to the inhibition of liver injury (92). RCJ and RCJ protected T2DM-associated NAFLD rats with metabolic disorders and liver damage by regulating bile acid enterohepatic circulation to maintain bile acid homeostasis, whereas the effect of RCJ was more prominent. The comparison of fermented and raw Cili juice in this study yielded further findings in contrast to those of previous scholars, who proposed that fermented Cili juice maintained bile acid homeostasis through enterohepatic circulation, whereas our study confirmed that the regulatory effect of RCJ on bile acid homeostasis was significantly stronger than that of FCJ (23). This disparity might be attributed to different fermentation methods.

Metabolic syndrome induced by a high-cholesterol diet significantly reduces cholesterol efflux capacity. Current research highlights that PPAR has been identified as a critical factor in the pathogenesis of both T2DM and NAFLD (93). Specifically, PPARγ may enhance the expression of apolipoprotein A1(APOA1), thereby promoting the molecular transport of insulin receptor substrate 1 (IRS1). This interaction forms the PPAR-APOA1 signaling pathway, which plays a pivotal role in the development and progression of NAFLD (94). In this study, the *Apoa2* transcript level was significantly lower in the T2DM group than in the control group, and the *Apoa2* level increased after treatment with Cili juice. In particular, RCJ can significantly increase the expression levels of *Apoa2* and *Apoc1* to increase the level of cholesterol efflux in T2DM-associated NAFLD rats. At the same time, the expression pattern of *Irs3*, a gene related to IR, followed a similar trend. In addition, we found that M-Cili-R was superior to M-Cili-F in reducing the transcription level of *Fabp5* in T2DM-associated NAFLD rats (*p* < 0.05), indicating that RCJ was more effective than fermented juice in inhibiting hepatic fat synthesis in T2DM rats. Similarly, *Cpt1b*, a rate-limiting enzyme in fatty acid β-oxidation, was significantly upregulated in the M-Cili-R group compared with the T2DM group (*p* < 0.05). In rats with T2DM-associated NAFLD, RCJ had better effects on fatty acid oxidation than did FCJ.

In addition, terpenoid synthesis and retinol metabolism were more significantly enriched in the M-Cili-F group than in the M-Cili-R group. The expression levels of the *Idil* and *Hmgcr* genes in T2DM-associated NAFLD rats were upregulated after intervention with Cili juices and were significantly upregulated in the M-Cili-F group (*p* < 0.01). IDI1 is an important factor affecting the final production of terpenoids. Studies have shown that the overexpression of IDI1 in yeast can increase the production of terpenoids, which protect the liver through anti-inflammatory, antioxidative, and antiapoptotic mechanisms and through the metabolic regulation of bile acid; thus, most traditional Chinese medicines for liver and gallbladder protection are rich in terpenoids with strong biological activity and low toxicity (95). The terpenoids in Cili can improve antioxidative stress and immunity. Dechang et al. reported that the co-fermented broth of Cili and edible fungus regulated the immunity of immunosuppressed mice (96). In addition, we found that retinol metabolism was significantly enriched in the M-Cili-F group, and the expression of *Cyp2c22*, an enzyme involved in retinoic acid metabolism in the liver, was downregulated after long-term intervention with Cili juices in T2DM model rats (*p* < 0.01). Studies have shown that interactions between insulin and vitamin A signaling systems regulate hepatic glucose and lipid metabolism, but few studies have investigated the effects of P450 superfamily enzymes on retinol metabolism in diabetes, and further experiments are needed to verify this hypothesis. In this study, the protection of FCJ against diabetes was related to the formation of terpenoids and retinol metabolism, which may have been regulated by the rich diversity of wild yeast species and their post fermentation products in FCJ. These findings may be related to immunomodulatory and antioxidant effects, which are worthy of further study.

In conclusion, though RCJ and FCJ share core bioactivities due to common components (e.g., quercetin, rhamnetin), FCJ exhibits superior efficacy in anti-inflammation, FCJ significantly elevated the urinary levels of anti-inflammatory substances in T2DM-associated NAFLD rats and markedly enhanced the liver’s capacity for synthesizing anti-inflammatory terpenoids. While RCJ exhibits superior efficacy in hepatic lipidomics, it uniquely reduces the levels of multiple carnitines in the urine of T2DM-associated NAFLD rats, enhances bile acid secretion, balances bile acid metabolism, and consequently regulates systemic lipid metabolism.

By integrating the analysis of FCJ/RCJ components and transcriptome DEGs, we identified highly relevant genes and components. Our findings revealed that *Abcc3* was significantly positively correlated with digalacturonic acid, L-isoleucine, D-mannose, and L-aspartic acid but negatively correlated with xylobiose and D-ribose. Notably, D-ribose serves as a significant marker for T2DM complications (97). Additionally, *Abcc3* and *Hmgcs1* were significantly positively correlated with cortisol, estrone sulfate, and salicylic acid in FCJ/RCJ and negatively correlated with 2-oxoglutaric acid. Both *Abcc3* and *Hmgcs1* play crucial roles in maintaining cholesterol homeostasis, whereas D-galacturonate/D-mannose may increase the level of ascorbic acid (98). GPT2, a gluconeogenic enzyme, is utilized as a blood biomarker for liver injury. In this study, the *Gpt2* transcript level was significantly lower in the RCJ intervention group than in the T2DM group. Research has indicated that silencing *Gpt2* in diabetic livers can mitigate hyperglycemia by reducing amino acid gluconeogenesis and that insulin can suppress the expression of *Gpt2* and other gluconeogenic genes (99). Joint analysis further revealed that *Gpt2* was significantly negatively correlated with 2-oxoglutaric acid, citric acid, and fumaric acid, all of which are involved in the tricarboxylic acid cycle.

For the integrated analysis of urine metabolomics and liver transcriptomics, we employed the O2PLS model to identify and screen for potential genes and metabolites. SCD, a key enzyme in fatty acid metabolism, has been demonstrated to prevent obesity and diabetes in HFD-fed mice, improving their lipid metabolic status and insulin sensitivity. Our study revealed that both FCJ and RCJ could downregulate *Scd* transcription levels in the livers of T2DM-associated NAFLD rats. Research has confirmed that PRLR modulates liver insulin sensitivity in mice (100). The RNA-seq results of this study demonstrated that both FCJ and RCJ are capable of increasing the transcriptional level of *Prlr* in the livers of T2DM rats. Bile acid secretion is associated with canine uric acid metabolism, and the expression of canine uric acid is elevated in T2DM, showing a positive correlation with SLC22A8 (101). We observed that both FCJ and RCJ could downregulate the *Slc22a8* gene, which is markedly upregulated in T2DM-associated NAFLD rats, potentially contributing to the improvement of bile acid homeostasis in Cili juice. CYP2C13 expression was elevated in response to liver injury, and we noted that the transcription levels of *Cyp2c13* were reduced following FCJ intervention. Aberrantly high expression of *Sult1e1* can result in sterol imbalance, inhibition of the liver X receptor, and inflammation (102). We found that FCJ suppressed the aberrantly high expression of *Sult1e1* in T2DM-associated NAFLD rats.

Moreover, in the O2PLS model-calculated metabolites, when Proto IX abnormally accumulates in the liver and skin of mice, the clinical manifestations are predominantly liver-related and include liver cell damage, necrosis, inflammation, and even severe liver dysfunction and cholestasis. In the alcoholic steatohepatitis model, quercetin offers protection against oxidative damage in alcoholic liver cells; however, Proto IX abrogates the protective effects of quercetin (67). In this study, Proto IX levels were markedly elevated in the urinary metabolites of rats with T2DM-associated NAFLD and were significantly reduced following the administration of FCJ and RCJ. The concentration of α-tocopheryl acetate, a potent antioxidant, was significantly elevated in the urine of T2DM rats following RCJ intervention. Moreover, the excretion of taurochenodeoxycholate in rats with T2DM-associated NAFLD through the urine was also greater in the RCJ intervention group than in the model group. Thus, the hepatoprotective effect of Cili treatment has been validated. The genes and metabolites identified through the O2PLS model serve as potential biomarkers for metabolomics and transcriptomics, indicating that the antidiabetic effects of FCJ and RCJ in rats with T2DM-associated NAFLD are mediated through improvements in insulin sensitivity, fatty acid metabolism, and bile acid homeostasis.

While this study provides compelling evidence for the therapeutic potential of FCJ/RCJ in T2DM-associated NAFLD, several limitations should be acknowledged. A key limitation lies in the sample size for omics analyses (*n* = 3/group), which may affect statistical generalizability. Future studies with expanded cohorts are needed to confirm these metabolic-transcriptomic network-level insights. In addition, our experimental design exclusively utilized male SD rats, precluding extrapolation of findings to female models. Future studies incorporating female rats will clarify gender-dependent intervention responses to FCJ/RCJ. Another limitation is that, owing to the constraints of the UPLC-MS approach, this research failed to detect the macromolecular compounds, polysaccharides, in FCJ and RCJ, and was more concentrated on the detection of small molecular compounds. The study of polysaccharides in Cili has been included in our subsequent research plan.

In conclusion, this study identified differentially abundant metabolites and differentially expressed genes in rats with T2DM-associated NAFLD following the consumption of FCJ and RCJ, revealing substantial alterations in their urinary metabolic profiles and hepatic transcriptome profiles. This study revealed that both FCJ and RCJ have antidiabetic, hypoglycemic, lipid-lowering, and hepatoprotective effects. Notably, FCJ has more pronounced anti-inflammatory effects, whereas RCJ has a stronger regulatory effect on bile acid homeostasis. These findings offer novel insights and evidence into the distinct metabolic pathways influenced by the spontaneous fermentation of Cili juice and raw Cili juice in diabetic rats. These results hold considerable importance for the prevention and management of T2DM-associated NAFLD and warrant further in-depth investigation.

## 5 Conclusion

In conclusion, both FCJ and RCJ have hypoglycemic, lipid-lowering, and hepatoprotective effects, long-term oral administration of raw and fermented Cili juice with T2DM-associated NAFLD could ameliorate hyperglycemia and dyslipidemia, reduce abnormal lipid accumulation in the liver, decrease abnormally elevated indicators of liver function and improve insulin expression in the pancreas. The antidiabetic effects of FCJ/RCJ in rats with T2DM-associated NAFLD are mediated through improvements in insulin sensitivity, fatty acid metabolism, and bile acid homeostasis. Among them, raw Cili juice had a more significant regulatory effect on lipid β-oxidation and bile acid homeostasis, whereas fermented Cili juice had a stronger anti-inflammatory effect. Our research suggests that the two kinds of Cili juices, as functional foods, may play protective roles in alleviating symptoms of glycolipid disorders and liver damage in T2DM-associated NAFLD.

## Data Availability

The data presented in the study are deposited in the SRA repository, accession number: PRJNA1259692.
