# Supplementary material for: Prevention of high-fat/high-sugar diet-induced type 2 diabetes mellitus-associated non-alcoholic fatty liver disease in rats with fermented and raw Rosa roxburghii Tratt (Cili) juice
Source: Front Nutr. 2025 May 19;12:1584551. doi: 10.3389/fnut.2025.1584551 (PMC12127171; doi:10.3389/fnut.2025.1584551)
Supplement: Supplementary file 1 [file Data_Sheet_1.pdf]

贵州醫科大學
GUIZHOU MEDICAL UNIVERSITY

动物实验伦理审查表

Animal Experimental Ethical Inspection Form of Guizhou Medical University

编号（No）：2101266

申请人填写的信息 (Related information filled by applicant)					
	申请单位<br>Name of organization		贵州医科大学医学检验学院		
	申请人<br>Applicant		潘卫	联系电话<br>Telephone	13511929839
	实验名称<br>Experiment title		基于“肠-肝轴”探讨刺梨对糖尿病肝损伤防治机制的研究		
	申请日期<br>Application date		2021 年 7 月 1 日		
	拟实验时间<br>Experiment date		2022 年 1 月至 2024 年 12 月		
	使用动物情况	品种品系<br>Species of strain	SD 大鼠	等级<br>Grade	SPF 级
		实验设施合格证编号<br>Reg. No. of Experimental Facilities certification	SYXK(黔)2018-0001	规格<br>Specifications	大鼠 180-220g;

实验要点：包括实验目的、实验方法、观测指标、实验结束后处死动物的方法等
Outline of experiments, including aim of experiment ， experimental methods, observational index, executing animal method, et al.

实验目的：本项目拟在糖尿病大鼠模型中，研究刺梨基于肠-肝轴对肝脏的保护作用，为阐释刺梨对糖尿病肝损伤的脂质代谢调控机制提供实验和理论依据。

实验方法：选取健康 SD 大鼠若干只，分为正常组、糖尿病模型组、刺梨实验组。高脂高糖饲料喂养复制糖尿病模型，并对刺梨实验组进行每日刺梨汁灌胃。达到实验所需时长后，取大鼠血液、肝组织、粪便进行脂质组学分析、肠道菌群测序分析及粪菌移植等实验。

观测指标：外周血检测肝肾功能、血糖、血脂指标，肝脏组织行 HE 染色、油红 O 染色；RT-PCR 检测相关基因 mRNA 水平，Western blot 检测肝脏损伤、脂质代谢的相关指标蛋白质表达水平等、粪便进行肠道菌群测序分析等。

实验结束后处死动物的方法：麻醉处死。

（请翻看背面）

<table>
<tr><td>申请者声明
Announcement of applicant</td><td colspan="2">我将自觉遵守实验动物福利伦理原则，随时接受实验动物伦理委员会的监督与检查，如违反规定，自愿接受处罚。
I will abide by the rules of animal experimental ethics, accept the supervision and inspection of the animal experimental ethics committee, and accept the punishment in case of any infringement.)

申请者签名：[illegible]
2021 年 7 月 1 日</td></tr>
<tr><td>审查依据
Inspection contents</td><td colspan="2">1. 该项目是否必须用实验动物进行实验，即能否用计算机模拟、细胞培养等非生命方法替代动物或用低等动物替代高等动物进行实验(Does laboratory animal must be used in the project? Could other methods such as computer simulation．cell culture or using the low-grade animal instead of the high－grade animal?)
2. 表中所填申请人资格和所用动物的品种品系、质量等级、规格是否合适，能否通过改良设计方案或用高质量的动物来减少所用动物的数量(Are the qualification of applicant，species or strain，grade and specifications of animals suitable? Could the quantity of animals be reduced by improving the study design or using high quality animals?)
3. 能否通过改进实验方法、调整实验观测指标、改良处死动物的方法，来优化实验方案、善待动物(Could the study design and animal treatment be refined by ameliorating experimental method，adjusting observational index．executing animal method?)</td></tr>
<tr><td rowspan="3">审查结果
Results of inspection</td><td>课题负责人意见
Project director attitude</td><td>同意。 签名：[illegible]</td></tr>
<tr><td>实验动物伦理委员会委员意见
Members attitude of the Animal Care Welfare Committee</td><td>同意。 签名：[illegible]</td></tr>
<tr><td>实验动物伦理委员会意见
Attitude of the Animal Care Welfare Committee</td><td>贵州医科大学 GuiZhou Medical University 实验动物伦理委员会 the Animal Care Welfare Committee
签章
2021 年 7 月 3 日</td></tr>
<tr><td colspan="3">备注:
Remark</td></tr>
</table>

说明：
1. 申请表审核结束后，一式 2 份递交到贵州医科大学实验动物中心盖章。
2. 课题负责人、执行人及合作单位负责人均需在申请者签字栏签字。
3. 需在外单位完成课题的，请同时填写校外实验动物设施使用证明。
4. 表格签名处必须手写。
5. 要求写明项目的意义、必要性、项目中有关实验动物的用途、饲养管理或实验处置方法、预期出现对动物的伤害、处死动物的方法、项目进行中涉及动物福利。
