# Supplementary material for: Prevention of high-fat/high-sugar diet-induced type 2 diabetes mellitus-associated non-alcoholic fatty liver disease in rats with fermented and raw Rosa roxburghii Tratt (Cili) juice
Source: Front Nutr. 2025 May 19;12:1584551. doi: 10.3389/fnut.2025.1584551 (PMC12127171; doi:10.3389/fnut.2025.1584551)
Supplement: Supplementary file 2 [file Data_Sheet_2.docx]

Supplementary Material

# Supplementary Figures and Tables

## High-fat-High-sugar-diet (HFD) for 12 weeks induced insulin resistance in rats

In the process of establishing the T2DM rat model, the experimental rats were first fed a high-fat diet for 12 weeks, after which the insulin resistance of the rats was detected, and an oral glucose tolerance test was performed to detect islet damage. When insulin resistance appeared, the next step of the experiment was to induce T2DM by the intraperitoneal injection of a small dose of STZ (25 mg/kg). In this study, we performed OGTT, insulin release tests and C-P release tests in rats fed a high-fat diet for 12 weeks. The rats were given 50% glucose (2 g/kg) orally after 12 hours of fasting. Blood samples were collected from the orbit at 0, 30, 60, 120 and 180 min after gavage (see **Supplementary Figure 1**). The HOMA-IR calculation formula (HOMA-IR=FBG (mmol/L) ×serum insulin level (mU/L)/22.5) confirmed that the rats fed a high-fat diet developed IR before further STZ injection to establish the T2DM disease model.


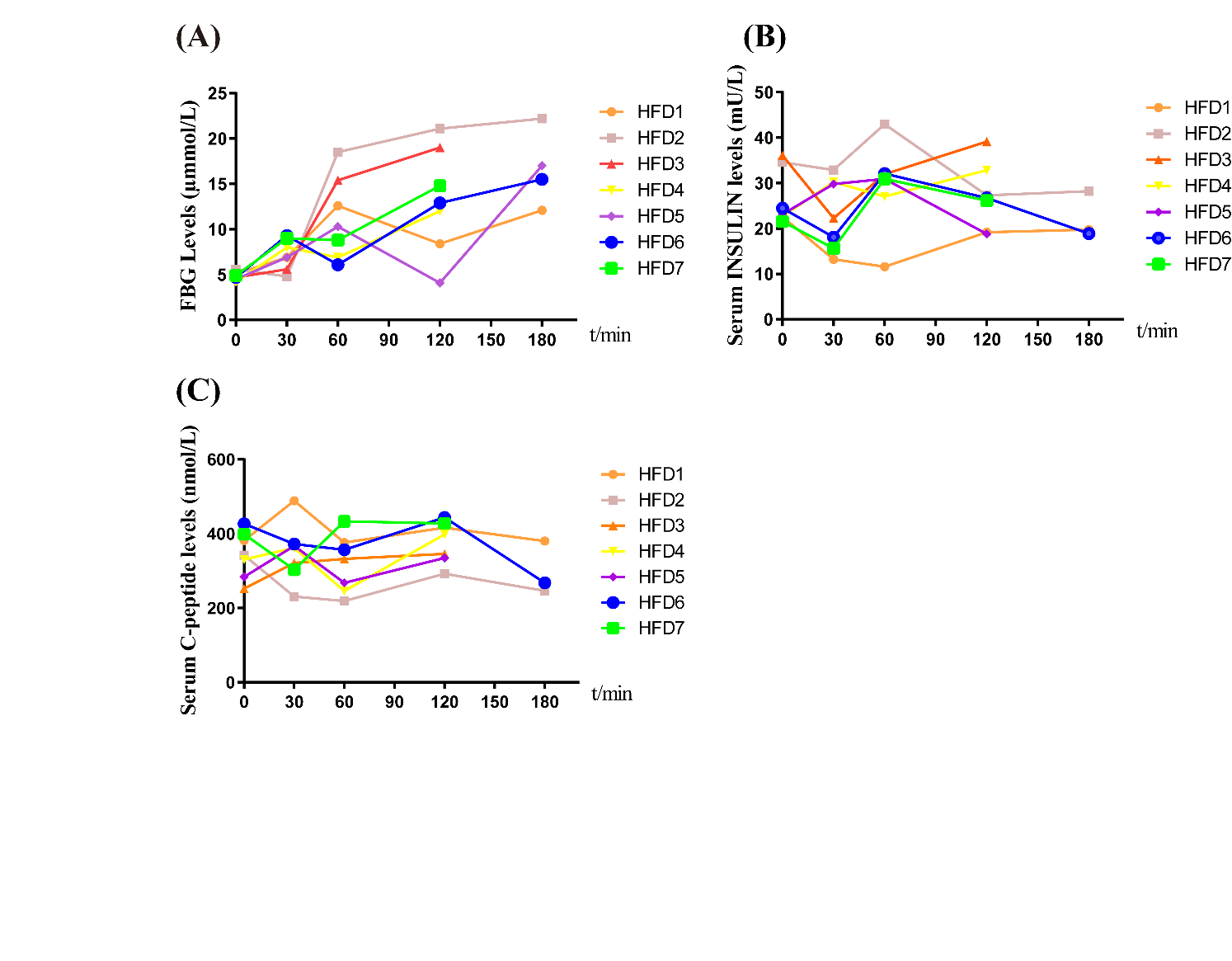


## Supplementary Figure 1. OGTT test, INSULIN release test and C-P Release test results of HFD rats. (A) FBG levels in the tail vein, (B) Serum INSULIN levels, (C) Serum C-Peptide levels. Data are presented as mean±SD (n=7).

## Preliminary experiment

In the pilot study, to determine whether fermented Cili juice and raw Cili juice would affect the body weight, blood glucose, blood lipid content and liver function of SD rats and to explore the appropriate dosage of Cili juice by gavage, we established 10 groups: the normal diet + normal saline group (NC), normal diet + low dose of Cili fermented juice group (NFL), normal diet + high dose of Cili fermented juice group (NFH), normal diet + low dose of Cili raw juice group (NRL), normal diet + high dose of Cili raw juice group (NRH), T2DM + normal saline group (T2DM), T2DM + low dose of Cili fermented juice group (T2FL), T2DM + high dose of Cili fermented juice group (T2FH), T2DM + low dose of Cili raw juice group (T2RL), and T2DM + high dose of Cili raw juice group (T2RH).

The low dose was 1.5 mL/kg/day, and the high dose was 3.0 mL/kg/day. Intragastric administration was performed daily at 6 PM after the rats had eaten for 20 weeks.

After 20 weeks of continuous intervention, as shown in **Supplementary Figure 2**, compared with those in the NC group, the body weight, FBG, TG, TC, ALT and AST indices of the experimental rats did not significantly differ between the NFL, NFH, NRL, and NRH groups, which suggested that the fermented Cili juice and raw Cili juice, as ordinary daily drinks, did not have the side effects of increasing blood glucose and damaging metabolic organs, indicating that Cili juice taken as a long-term drink.

Compared with those in the T2DM group, the levels of FBG, TG, TC and ALT in the T2FL, T2FH, T2RL and T2RH groups improved after 20 weeks of Cili juice intervention, and the differences were statistically significant (*p*<0.05). The high dose of Cili raw juice group (T2RH) increased the body weight of T2DM rats (*p*< 0.05), and both the high dose of Cili fermented juice (T2FH) and raw juice (T2RH) groups presented significantly lower AST levels than did the T2DM group (*p*<0.05). The observation of peripheral blood biochemical indicators and body weight revealed that both high and low doses of Cili juice improved the indices of dysglycemia, dyslipemia and liver injury in T2DM rats. Moreover, we recorded the survival probability of the T2DM rats in each group after 20 weeks of Cili juice intervention (see **Supplementary Figure 2G**). The initial number of rats in each group was 14. As shown in the figure, the survival rate of the rats in the T2DM model group decreased gradually over time, and only approximately 20% of them survived at 20 w. Compared with the T2DM groups, the interventions of both Cili fermented juice and Cili raw juice demonstrated greater ability to improve the survival rate of T2DM-associated NAFLD rats Based on the aforementioned pre-experimental findings, it has been demonstrated that both FCJ and RCJ exert beneficial effects on improving biochemical parameters and enhancing the survival rate of T2DM rats. Notably, low-dose interventions have already exhibited significant efficacy in T2DM-associated NAFLD rats. In light of economic considerations and adherence to the "3R principle" for animal welfare (reducing the intragastric administration dose and minimizing the number of experimental animals), a low intragastric dose of 1.5 mL/kg/day was selected for subsequent experiments.


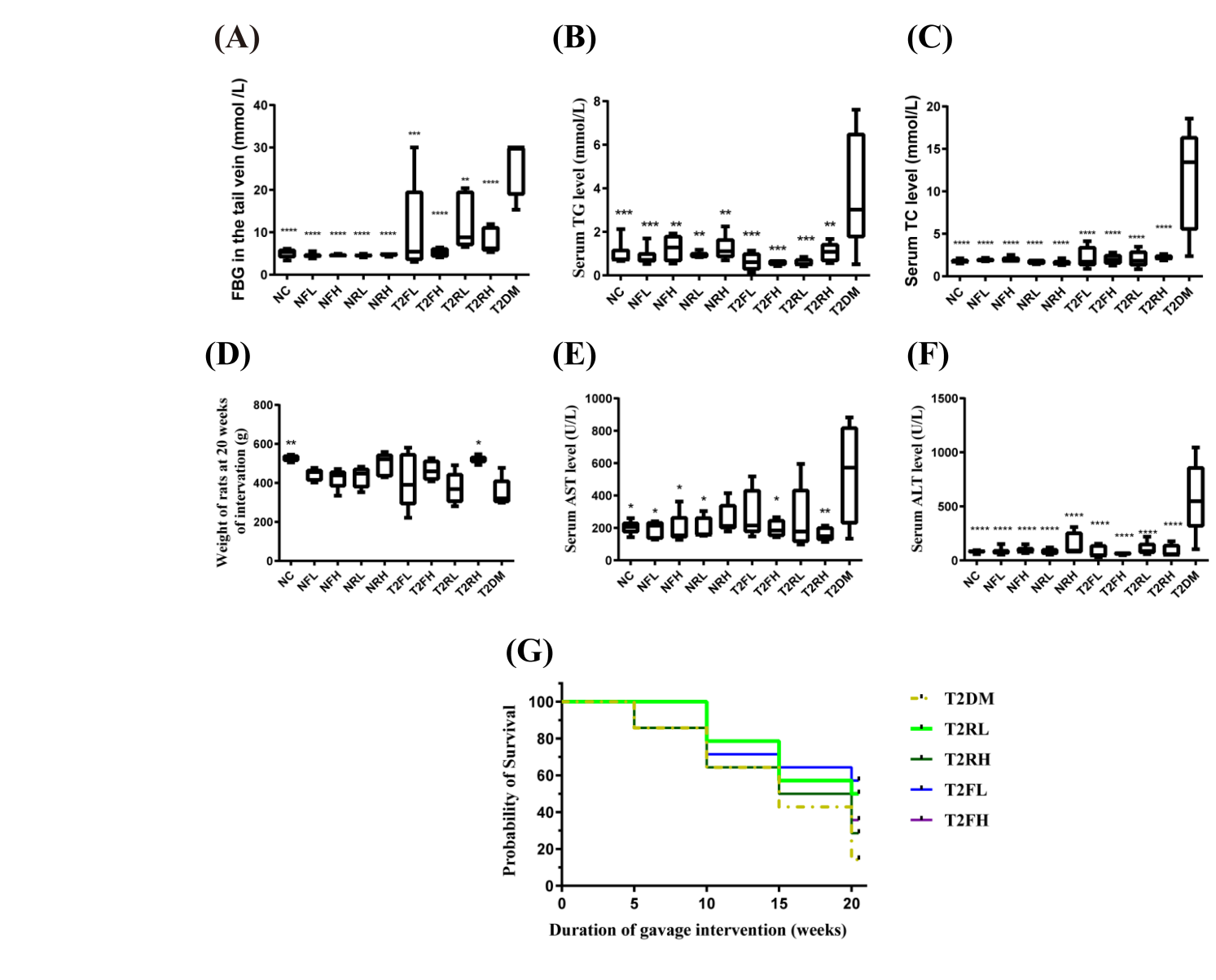


**Supplementary Figure 2.** Biochemical indices of the rats in each group in the preliminary experiment. (A) FBG levels in the tail vein, (B) Serum TG levels, (C) Serum TC levels, (D) Weight of rats in 20 weeks of intervention, (E) Serum AST levels, (F) Serum ALT levels. Data are presented as mean±SD (n=4-6). (G) Probability of Survival in 20 weeks (initial number of rats in each group was 14). **p*＜0.05，***p*＜0.01，****p*＜0.001, compared with T2DM group.

## Supplementary figure for UPLC/MS analysis of FCJ and RCJ

##
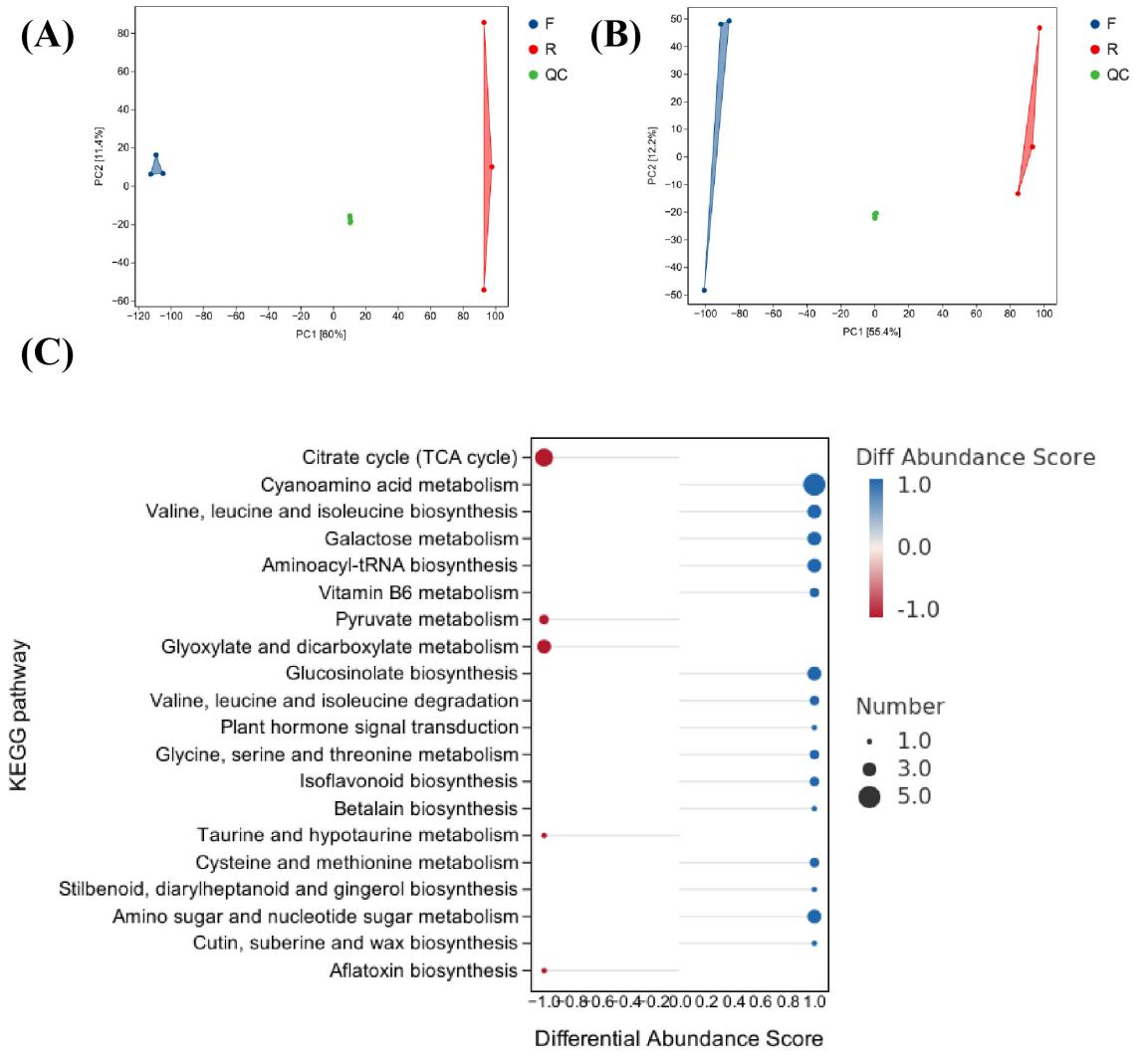


## Supplementary Figure 3. Supplementary Analysis of differential components in FCJ and RCJ. (A-B) PCA diagrams of FCJ, RCJ and QC in anion and cation modes, respectively, (C) KEGG pathway enrichment analysis of FCJ vs. RCJ in both anion and cation modes.

## Supplementary figure for Urine Metabolism and Liver RNA-seq


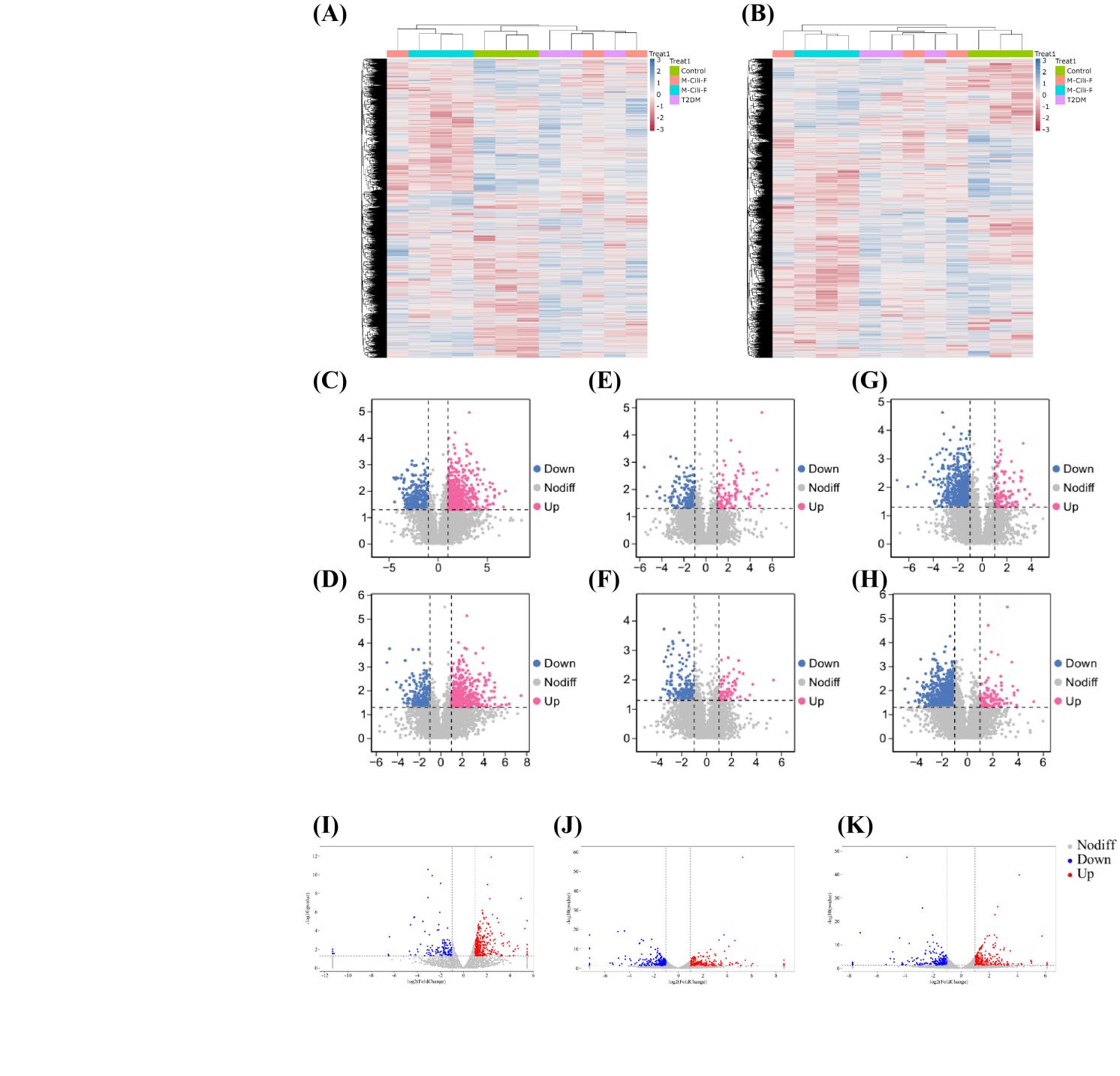


## Supplementary Figure 4. Supplementary Analysis of Urine Metabolism and Liver RNA-seq in Rats. (A) Heatmap of overall substance clustering in anionic mode of urine metabolism. (B) Heatmap of overall substance clustering in cation mode of urine metabolism, C-H were differentially metabolized compounds in the four groups of rat urine. (C-D) Volcano plots 1052nof the control and T2DM groups in negative mode and positive mode. (E-F) Volcano plots of the T2DM and M-Cili-F groups in negative mode and positive mode. (G-H) Volcano plots of the T2DM and M-Cili-R groups in negative mode and positive mode. I-K were DEG analysis of different groups of rats with RNA-seq. (I) Volcano plots of DEGs in the control vs. T2DM, (J) Volcano plots of DEGs in the T2DM vs. M-Cili-F, and (K) Volcano plots of DEGs in the T2DM vs. M-Cili-R groups.

## qRT-PCR Primer

| No. | Name（*Ra*t） | Forward sequence (5’-3’) | Reverse sequence (5’-3’) |
| --- | --- | --- | --- |
| 1 | *Cyp51* | ACATAGCCCACTTCAAGCAGTAT | CCTTCTCGTTGAGCTGACTTCTA |
| 2 | *Cyp7a1* | GCTTTCCAGTGCATCCTTGAATA | GAAAGTCAAAGGGTCTGGGTAGA |
| 5 | *Cyp7b1* | CAGATGCAAAGACGGTCAGAAAT | AGCCAAGATGATGTGCTCCTATT |
| 7 | *Irs3* | CGCCACCTGATCGTCATCTATAC | GGGCGAAGATCCAAGACTCAG |
| 8 | *Abcc3* | ACTCTCACGTGGCTAAGCATATC | CCGTCAGCAAGCACAATGATAAA |
| 9 | *Pparγ* | ACCCTTTACCACGGTTGATTTCT | AGGCTCTACTTTGATCGCACTTT |
| 10 | *Cpt1b* | ATGTAAGTGACTGGTGGGAAGAG | GCTTGTTGGCTCGTGTTCTTAAT |
| 11 | *Idi1* | AGGACTACGCTAGATTGGCAATT | CTAGAACACAGCGATTCCAGAGA |
| 12 | *Fabp5* | GCACCTTGGGAGAGAAGTTTGAT | ACCTTCTCATAGACCCGAGTACA |
| 13 | *Fdft1* | TGCTGCGAAACTTTCACACTTTC | AGTACTTGTCCCAGTCCTGTTTG |
| 14 | *Ugt1a6* | TTGGTCATCCAAAGGCTCGG | GGCGCATGATGTTCTCCTTG |
| 15 | *Hsd17b7* | TTATACCTGAACGCCGGAATCAT | TGGAACCCATCAGCAGTAATCTT |
| 16 | *Apoc1* | GCAATGGAGAGCTTACCGGATAA | AACCAGTTCCGAGTCTTGATCAT |
| 17 | *Apoa2* | CTGTTCAGCCAGTATCTTCAGAGT | CATCAGATTCGTCCCAGTTCTCT |
| 18 | *Slc27a5* | GCAACGTGGGCTTAATGAACTAT | CTCTGCCGTCTCTATGTCAAACT |
| 19 | *Hmbs* | ACCCTTGTGATGCTGTTGTT | GTGTTGAGGTTTCCCCGAAT |

**Supplementary Table 1.** Primer sequences

The reaction procedure was predenaturation at 95℃ for 30 s. PCR reaction (95 ° C for 5 s,

60℃ for 30 s) x40 cycles; Melting curve (45℃~ 90℃, Increment 0.1ºC/s).
